# An enhancer trap system to track developmental dynamics in Marchantia polymorpha

**DOI:** 10.1111/tpj.16394

**Published:** 2023-08-15

**Authors:** Alan O. Marron, Susanna Sauret‐Güeto, Marius Rebmann, Linda Silvestri, Marta Tomaselli, Jim Haseloff

**Affiliations:** ^1^ Department of Plant Sciences University of Cambridge Downing Street Cambridge CB2 3EA UK; ^2^ Present address: Crop Science Centre University of Cambridge 93 Lawrence Weaver, Road Cambridge CB3 0LE UK

**Keywords:** *Marchantia polymorpha*, enhancer trap, Gemma, Rhizoid, Oil Cell, margin tissue, meristem, auxin, sporeling

## Abstract

A combination of streamlined genetics, experimental tractability and relative morphological simplicity compared to vascular plants makes the liverwort *Marchantia polymorpha* an ideal model system for studying many aspects of plant biology. Here we describe a transformation vector combining a constitutive fluorescent membrane marker with a nuclear marker that is regulated by nearby enhancer elements and use this to produce a library of enhancer trap lines for Marchantia*.* Screening gemmae from these lines allowed the identification and characterization of novel marker lines, including markers for rhizoids and oil cells. The library allowed the identification of a margin tissue running around the thallus edge, highlighted during thallus development. The expression of this marker is correlated with auxin levels. We generated multiple markers for the meristematic apical notch region, which have different spatial expression patterns, reappear at different times during meristem regeneration following apical notch excision and have varying responses to auxin supplementation or inhibition. This reveals that there are proximodistal substructures within the apical notch that could not be observed otherwise. We employed our markers to study Marchantia sporeling development, observing meristem emergence as defining the protonema‐to‐prothallus stage transition, and subsequent production of margin tissue during the prothallus stage. Exogenous auxin treatment stalls meristem emergence at the protonema stage but does not inhibit cell division, resulting in callus‐like sporelings with many rhizoids, whereas pharmacologically inhibiting auxin synthesis and transport does not prevent meristem emergence. This enhancer trap system presents a useful resource for the community and will contribute to future Marchantia research.

## Significance Statement

The liverwort *Marchantia polymorpha* is an emerging model species with huge potential for synthetic biology. We generated an enhancer trap library as a resource for Marchantia research and characterized marker lines for several important cell types (rhizoids, thallus margin, apical notch and oil cells). These marker lines can be used to track cellular dynamics in growing plants, exemplified by studying auxin responses and sporeling growth, revealing new insights into thallus development and cell fate specification.

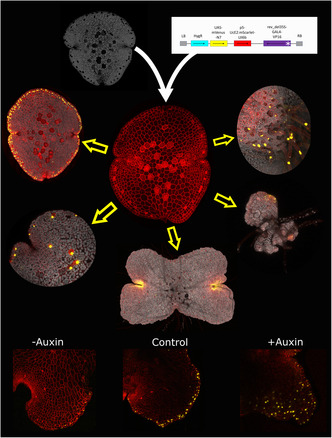

## INTRODUCTION

The liverwort *Marchantia polymorpha* has emerged as an increasingly important model system with which to study plant evolution, development and molecular biology (Bowman et al., 2017; Shimamura, 2016). There are established protocols for genetic manipulation of Marchantia sporelings by Agrobacterium, which can yield thousands of transformants per transformation experiment (Ishizaki et al., 2016; Sauret‐Güeto et al., 2020; Sugano et al., 2014). The haploid gametophyte is the dominant phase of the life cycle, which makes phenotypic characterization straightforward as recessive alleles are not a limitation. Liverworts occupy an important phylogenetic position within the land plant phylogeny, forming the bryophyte clade together with mosses and hornworts (Harris et al., 2020; Puttick et al., 2018; Su et al., 2021). Sequencing the Marchantia genome revealed that it is highly streamlined, with relatively low levels of genetic complexity and no evidence for whole genome duplication events, unlike mosses (Bowman et al., 2017; Rensing et al., 2007, 2020). Liverworts have a degree of morphological complexity, with a variety of tissue and cell types (Shimamura, 2016).

The plants themselves have fast growth on defined agar media and controlled growth conditions and can be propagated asexually by excising fragments of the thallus. Furthermore, Marchantia thalli produce clonal propagules (gemmae) which arise from a single progenitor cell within a gemma cup, meaning that clonal lines can be easily established after only a few weeks. Gemmae have a stereotypical pattern of growth and germinate almost immediately after removal from a gemma cup. Growth proceeds in an open fashion and is directly accessible for observation by live microscopy. The gemma itself is a roughly ovoid flattened sheet of cells, with a stalk scar at the former point of attachment to the gemma cup, and two apical notches approximately perpendicular to this on either side of the gemma. In the centre of the gemma, there are numerous rhizoid precursors; large cells that are polygonal in shape (Shimamura, 2016). Running around the periphery of the gemma, and dotted through the central region of the thallus, are oil cells. Oil cells contain membranous organelles called oil bodies, which produce secondary metabolites that provide anti‐herbivore defense (Kanazawa et al., 2020). The edge of the gemma is only one cell layer thick, however, the gemma is thicker further towards the centre where parenchyma tissue is enclosed between two layers of epidermal tissue.

The initial phase of gemma germination sees the rapid emergence of rhizoids on both the dorsal and ventral side of the gemma. Initially, the gemma has no dorsoventral asymmetry, with this axis being established after 48 h due to gravitation cues (Bowman et al., 2016; Miller & Voth, 1962). After this, the ventral side features rhizoids (pegged and smooth), and mucilage papillae (Shimamura, 2016). Cell division contributes to rapid gemma growth, and occurs at the meristematic areas at the apical notches (Taren, 1958). As cells are displaced outwards from the notch region they cease division and begin expanding, eventually reaching a mature state. After 3–5 days a split appears in the thallus around the notch, forming dorsoventral ‘flaps’ of thallus. After this split, air chambers and pores begin to appear (Apostolakos & Galatis, 1985). These repeating structures are formed by stereotypical patterns of divisions, which create an open space lined by photosynthetic filaments. The chamber is connected to the external environment by an air pore bounded by elongated, curved pore cells. Oil cells are found at various locations between the air pores. After approximately 7 days, bifurcation of the apical notches produces the branched thallus morphology (Solly et al., 2017), and after 2–3 weeks gemma cups appear on the thallus midrib (Kato, Yasui, & Ishizaki, 2020).

The combination of streamlined genetics, clonal growth, experimental tractability and moderate morphological complexity makes the Marchantia gemma an ideal system with which to study plant development. Gemmae have already been employed to examine processes as diverse as meristem regulation (Hirakawa et al., 2019, 2020), auxin signalling (Eklund et al., 2015; Flores‐Sandoval et al., 2015; Suzuki et al., 2021), ABA action (Eklund et al., 2018), rhizoid growth (Thamm et al., 2020), secondary metabolism (Takizawa et al., 2021), organelle dynamics (Kimura & Kodama, 2016), morphogenesis (Furuya et al., 2018) and light signalling (Inoue et al., 2019). In addition to this, Marchantia gemmae have a high capacity for regeneration, providing a further method for creation and propagation of transgenic lines (Tsuboyama‐Tanaka et al., 2015). Marchantia gemmae are a promising chassis for synthetic biology, to use it to design methods for engineering plant growth and synthesis of useful molecules. Important advances have been made in this field through the development of methods for chloroplast transformation (Frangedakis et al., 2021), modular modification of the Marchantia auxin signalling machinery (Kato, Mutte, et al., 2020), and the regulation of oil body formation (Kanazawa et al., 2020). A better understanding of the processes and patterns underlying gemma development would greatly boost this research. New cell and tissue‐specific reporters are required to highlight dynamic changes in gene expression, cell identity and specification of tissues. Marker systems developed for gemmae can also be employed for other aspects of the Marchantia life cycle, such as sexual structures, the diploid sporophyte or spore germination (Shimamura, 2016).

One powerful method for generating markers is to use an enhancer trap scheme. This involves designing a construct that fuses a reporter gene (e.g. GUS, *lacZ*, *GFP* etc.) to a minimal promoter sequence. The minimal promoter sequence typically comprises a TATA box and transcription start site, which is insufficient to drive the reporter gene expression by itself. The construct is then transformed into the study organism (e.g. by Agrobacterium‐mediated T‐DNA insertion) and becomes incorporated at a random location within the genome. If the site of insertion is close enough to a regulatory sequence [an enhancer or promoter‐proximal element (Blackwood & Kadonaga, 1998)] then this drives expression of the reporter gene. The phenotype can then be examined, to see when, where and under what physiological conditions the signal appears. This basic enhancer trapping technique has been further elaborated and improved, for example by incorporating the GAL4‐UAS transcriptional activator system (Brand & Perrimon, 1993; Engineer et al., 2005; Haseloff, 1999).

Enhancer trap systems were first developed in Drosophila (O'Kane & Gehring, 1987) and have since been used in a range of animal systems, such as mice (Shima et al., 2016) and zebrafish (Trinh & Fraser, 2013). In plants, however, enhancer trap approaches are especially powerful to track cell fates, given the flexibility of plant cellular identity and importance of positional cues over lineage effects (Scheres, 2001; Van den Berg et al., 1995; Yu et al., 2017). Enhancer trap libraries have been produced for several plant systems, most notably Arabidopsis (Campisi et al., 1999; Haseloff, 1999; Klimyuk et al., 1995; Laplaze et al., 2005; Radoeva et al., 2016; Sundaresan et al., 1995), but also rice, lotus, tomato and moss (Buzas et al., 2005; Hiwatashi et al., 2001; Johnson et al., 2005; Pérez‐Martín et al., 2017; Wu et al., 2003). In Arabidopsis, enhancer trap methods have been particularly useful for marking cell and tissue types in systems with tightly defined growth and developmental dynamics, such as in roots (Zhang et al., 2019) or stomatal guard cells (Gardner et al., 2009) or for examining gene expression under specific physiological conditions (Baxter‐Burrell, 2003). Enhancer trap systems have also yielded new mutant phenotypes and allowed the identification of novel gene functions, for example in tomato fruit development (Pérez‐Martín et al., 2017). Enhancer trap lines can mark phenotypes that could not otherwise be easily accessible through conventional, loss‐of‐function genetic screens, for example, due to lethal effects of disrupting gene activity, or cryptic mutant phenotypes (Rojas‐Pierce & Springer, 2003). In such situations, locating the site of insertion within the genome can identify new gene functions and important novel promoter elements, that can then be used in further research (Radoeva et al., 2016; Román et al., 2020).

Here we further extend the use of enhancer trapping to a new plant group, the liverworts, by describing the production of an enhancer trap library in Marchantia and using it to investigate early gemma growth. Our enhancer trap construct includes a constitutive membrane marker to outline individual cells, allowing for easier identification of cell types, recognition of individual marked cells, and from this tracking of cell divisions and cell lineages. The fast growth, open development and ease of imaging fluorescent protein marker signals *in vivo* were ideally suited to the enhancer trap approach. The work produced new markers for both easily recognizable cell types (rhizoids, oil cells) and poorly understood cellular processes (specification of the meristem/notch region during gemma development, sporeling growth and thallus regeneration). It also allowed the identification and characterization of a distinct tissue type, consisting of cells found running around the edge of the gemma. These markers were useful to study other developmental processes, such as spore germination and growth responses to auxin treatment. The establishment of this library in Marchantia provides new resources for future research.

## RESULTS AND DISCUSSION

### Generating and screening the Marchantia enhancer trap library

Sporelings were transformed with either the ET238 or ET239 enhancer trap vector, following an established protocol (Sauret‐Güeto et al., 2020) and then selected on media containing hygromycin. Sporelings were picked out at random and these T0 plants were grown until gemma cups formed. Gemmae were isolated, germinated and the G0 plants were monitored for nuclear mVenus signal appearing in the first 7 days.

The full‐screen involved transformation of sporelings with either the ET238 or ET239 vector, as both constructs showed equally promising results in pilot experiments. Having alternative constructs will allow for flexibility in reducing potential fluorescence emission spectra cross‐talk in downstream imaging experiments or for crossing enhancer trap lines with lines containing other fluorescent markers. Equal volumes (150 μl per transformation) of starting spore suspension taken from the same pooled sterilized sporangia extracts were used for each construct in the full screen. After 10 days, transformed sporelings were selected at random, picked out and 2 days later were observed by stereomicroscope to identify sporelings with observable fluorescent reporter signals (mVenus or eGFP/mScarlet as appropriate). These T0 plants were isolated for further analysis. In total 101 ET238‐construct sporelings and 353 ET239‐construct sporelings were taken to this stage. Isolated sporelings were then monitored for fluorescent reporter signals; plants with fluorescent signals were retained and the general distribution of signal noted (e.g. widespread, partial/chimeric, etc.); those plants with no observable signal were disposed of. It should be noted that plants without signals may not simply be due to transformation failures or silencing; it is possible that fluorescent signals would be observed under different growth conditions or at different developmental stages than T0 sporelings of this age. After this stage, 49 ET238 plants and 190 ET239 plants remained.

Approximately 2–3 weeks after isolation gemma cups appeared on these T0 plants. Where there were indications of chimeric transformants in the T0 generation, gemmae (G0) were only taken for screening from gemma cups that developed within parts of the plant displaying consistent signals. G0 gemmae were screened across 7 days. Only lines with nuclear mVenus reporter signal observed within this timeframe were retained after this screening phase: 32 ET238 lines and 125 ET239 lines. These lines were maintained in culture with some lines subsequently disposed of due to problems with fungal contamination or loss of expression due to gene silencing. In total, 26 ET238 lines were cultured with 25 cryopreserved and 122 ET239 lines were cultured with 121 cryopreserved. The statistics for each screening phase are given in Table 1. The percentages of selected plant lines that display reporter signals are broadly in line with enhancer trap projects from other plant species, such as Arabidopsis (48%), rice (26–59%), tomato (32–43%) and moss (30%) (Hiwatashi et al., 2001; Pérez‐Martín et al., 2017; Sundaresan et al., 1995; Wu et al., 2003).

**Table 1:** Statistics on the number of lines at each screening step

Screening stage	ET238	% ET238	ET239	% ET239
Sporelings isolated	101	100%	353	100%
Isolated sporelings with mVenus signal	49	49% (100%)	190	54% (100%)
G0 gemma with mVenus signal by 7 dpg	32	32% (63%)	125	35% (66%)
Plants currently in culture or cryopreserved	26	26% (53%)	121	34% (64%)

Numbers are expressed as a percentage of sporelings isolated or isolated sporelings with signal (number in brackets). All observations were made by fluorescence stereomicroscope and “mVenus signal” refers to the presence of consistent nuclear reporter expression that was observable in multiple gemmae replicates.

mVenus expression was observed through stereomicroscope screening of gemma 1 day after removal from the gemma cup (i.e., 1 dpg) until 7 days after germination (7 dpg) in the lines that were maintained in culture (see Table 2). These signal patterns were recorded from multiple replicates from each line. Signal types were categorized according to mVenus expression in certain structures, cell types or spatial localization, and if this association was consistent across replicates. Within these broad categories of expression pattern, there did exist minor variability between gemmae from the same line, or occasional degree stochastic expression in individual cells. This is possibly due to leaky expression, cryptic variation in growth conditions, the typical morphological variability in Marchantia and the general complexity of enhancer activity across different cell and tissue types in multicellular organisms (Jenett et al., 2012). The signal patterns listed in Table 2 were subject to detection biases in favour of more obvious localization patterns (e.g. in the notch region), signals appearing in more easily recognizable cell types (e.g. oil cells, rhizoids), and in more prevalent or prominent morphological features (e.g. air chambers, distribution across the gemma centre or margins).

**Table 2:** Numbers of lines with expression patterns in selected morphological features

Expression pattern in 0 –7 dpg gemma	Number of lines
Meristem/apical notch	30
Oil cells	5
Rhizoid‐specific	13
Ventral surface but not rhizoids	1
Air chambers and air pores	38
Thallus periphery	2
Central zone	3
Absent from the central zone	3
Subepidermal tissues	2
Bulge behind the apical notch	2
Widespread/ubiquitous expression	11

Expression pattern means the areas or cell types where nuclear mVenus signal was observed by stereomicroscope in the first week after germination. The listed patterns had strong expression, were exclusive to or clearly concentrated in the indicated feature, were consistent across multiple replicate gemmae and persisted through the entire observation period. Other lines are present in the enhancer trap library that mark other morphological features, or have expression patterns in these morphological features but with weaker expression or non‐exclusive/concentrated distribution.

Notwithstanding this, we selected some examples to exemplify the utility of the enhancer trap approach in Marchantia. These lines had clear, reliable signals that appeared consistently and closely associated with certain cell types or thallus regions, with small amounts of variation between individual gemma. These lines were characterized for growing gemmae from 0 dpg to 3 dpg and then used for further studies, such as molecular characterization or response to phytohormone treatments.

### Rhizoids

Numerous enhancer trap lines showed nuclear mVenus signal in the dorsal rhizoids and/or rhizoid precursor cells. These cell types are easily recognizable, are present early in gemma development and contain large, prominent nuclei, making fluorescence relatively easy to observe. Table 3 lists the various types of rhizoid signals that were observed. Seven enhancer trap marker lines were specific to rhizoid‐type cells present in all rhizoids (dorsal and ventral), while a further six lines had signals specific to rhizoid‐type cells and the ventral surface of older gemma. It is noteworthy that no lines were found with dorsal rhizoid‐specific expression, supporting the notion that the dorsal rhizoids of early germinating gemma are not a unique cell type and that the gemma initially lacks fully differentiated dorso‐ventral asymmetry (Bowman et al., 2016; Miller & Voth, 1962). Early rhizoid signals were often seen in addition to nuclear signals being observed in other cells in the thallus, but this did not persist in the older thalli where only ventral rhizoids were growing, and mVenus marker expression was often irregularly distributed in some but not all rhizoids in the early gemma.

**Table 3:** Numbers of lines with various rhizoid‐related reporter signal

Nuclear signal distribution	No. of lines	No. of rhizoid and ventral surface‐specific lines	No. of rhizoid‐specific lines
All rhizoids	27	2	7
Dorsal, not ventral	16	0	0
Ventral, not dorsal	9	2	0
Both types but not in all rhizoids	18	0	0
Ventral only, but not all ventral rhizoids	27	2	0

Marker expression was observed by stereomicroscope in gemmae up to 7 dpg, lines were included if rhizoid signal was observed in some but not all of this period. For the 13 lines with rhizoid‐specific or rhizoid and ventral surface‐specific expression, fluorescent patterns were observed across multiple replicates and expression was persistent throughout the observation period. The ventral rhizoid classification includes both smooth and pegged rhizoid types.

**Figure 1:**
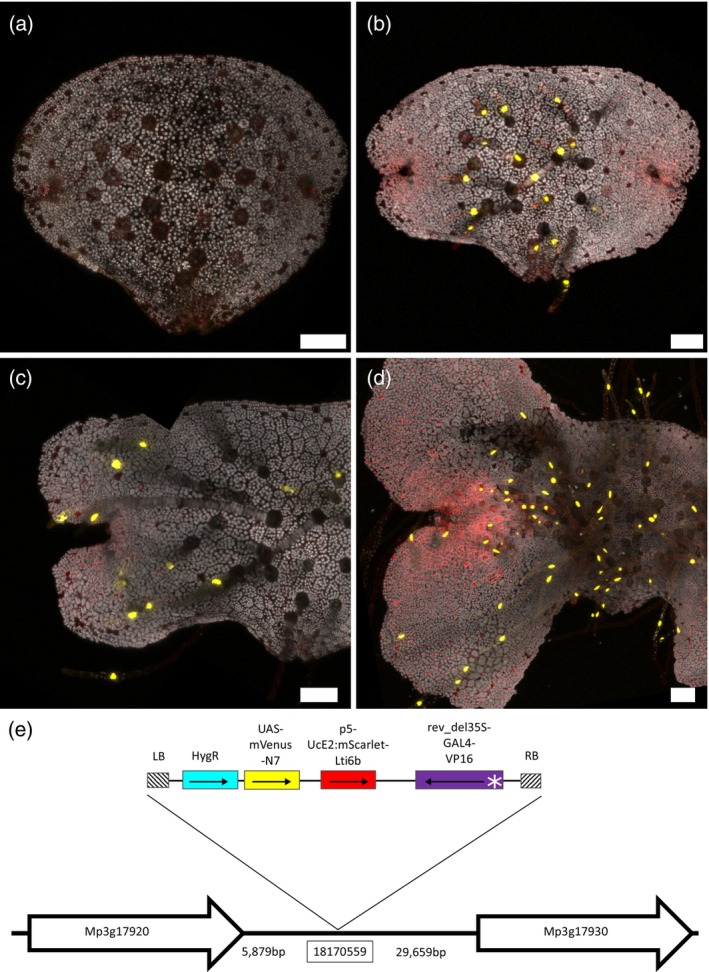
Rhizoid marker line ET239‐P177. The same gemma was imaged on 0 dpg (a), 2 dpg (b) and 3 dpg (c), showing localization of the mVenus marker signal to the rhizoid cells. (d) shows the ventral side of a 6dpg gemma, demonstrating that the marker signal is unique to rhizoids and is present in all rhizoid types. Scale bars = 100 μm. (e) schematic illustrating the genomic location of the insert site on chromosome 3. A possible candidate gene nearby is Mp3g17930 (Mapoly0039s0003), the Marchantia homolog of the root‐associated transcription factor RSL6. Neighbouring genes are indicated as white arrows, starting at the gene's start codon and pointing towards the gene's stop codon. Schematics are not drawn to scale and distances are indicated in base pairs. The T‐DNA map shows the relative positions of the minimal promoter (white asterisk), GAL4 enhancer (purple), mScarlet membrane marker (red), UAS‐mVenus nuclear marker (yellow) and hygromycin resistance gene (cyan), as well as the left and right border sequences (hatched). Black arrows point from the gene's start codon towards the stop codon.

Line ET239‐P177 was selected as a representative rhizoid‐specific marker line. It marked all rhizoid types: dorsal and ventral, pegged and smooth, as well as rhizoid precursor cells in the 0dpg gemmae, but not non‐rhizoid cells on the ventral surface of older gemmae. Confocal scanning microscope images of the marked rhizoids in this line are shown in Figure 1(a–d). The insertion site in line ET239‐P177 was identified as being at an intergenic site on chromosome 3 (18 170 559 in the MpTakv6.1 genome annotation). A putative candidate gene near this site is Mp3g17930 (Mapoly0039s0003) (Figure 1e), annotated in the *Marchantia polymorpha* v6.1 genome as being the Marchantia homolog of *Root Hairless‐like* 6. This is a transcription factor previously identified as being crucial for rhizoid development in liverworts and root development in vascular plants (Proust et al., 2016). This would tally with the observations of the rhizoid marker signal in line ET239‐P177, notwithstanding the uncertainties related to identifying the gene whose enhancer element has been trapped. Mp3g17930 does not appear in any of the identified scRNA‐seq data, but its UMAP plot does show some expression within the rhizoid cluster (Wang et al., 2023). ET239‐P177 therefore represents a line from this enhancer trap library marking a known cell type with obvious morphology and is likely linked to a gene previously known to be related to the development of that cell type.

### Oil cells

**Figure 2:**
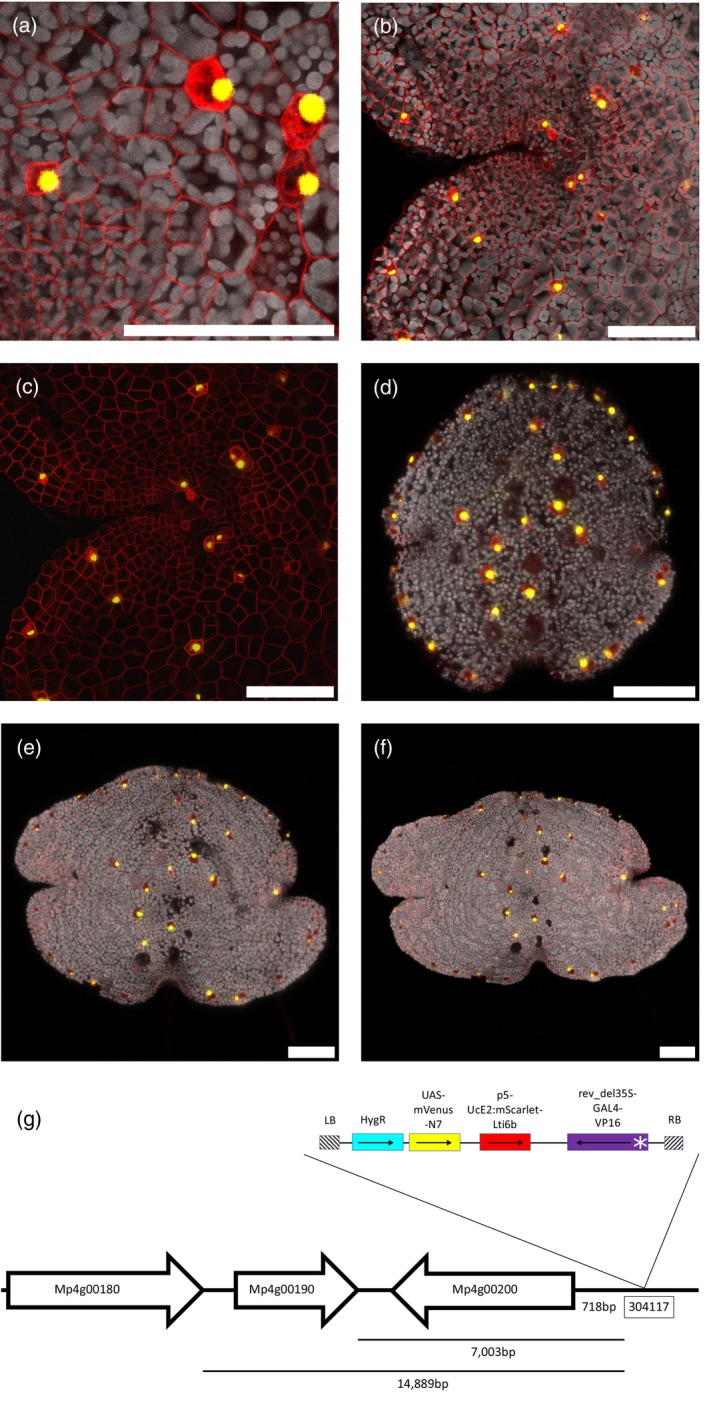
Oil cell marker line ET239‐P54. (a) The mScarlet membrane marker highlights the dense membranes of the oil bodies. (b) and (c) show a close‐up of a portion of a 3 dpg gemma with (b) and without chlorophyll autofluorescence (grey) (c), demonstrating that the marker signal in ET239‐P54 is unique to the oil cells. The same gemma was imaged at 0 dpg (d), 2 dpg (e) and 3 dpg (f), showing localization of the mVenus marker signal to the oil cells. Scale bars = 100 μm. (g) illustrates the genomic location of the insert site on chromosome 4, near possible candidate WRKY family transcription factor genes Mp4g00200 (Mapoly0162s0001) and Mp4g00180 (Mapoly0162s0003). Neighbouring genes are indicated as white arrows, starting at the gene's start codon and pointing towards the gene's stop codon. Schematics are not drawn to scale and distances are indicated in base pairs. The T‐DNA map shows the relative positions of the minimal promoter (white asterisk), GAL4 enhancer (purple), mScarlet membrane marker (red), UAS‐mVenus nuclear marker (yellow) and hygromycin resistance gene (cyan), as well as the left and right border sequences (hatched). Black arrows point from the gene's start codon towards the stop codon.

Oil cells are a cell type containing oil bodies (a membrane‐rich organelle containing various secondary metabolites including terpenoids and (bis‐)bibenzyl compounds) found throughout the Marchantia thallus (Kanazawa et al., 2020). Identification of oil cells depends on recognizing these oil bodies [clearly distinguishable with a membrane marker, Figure 2(a)] and on other features such as having fewer and smaller chloroplasts. In the 0dpg gemma, oil cells are found at regular intervals around the circumference of the gemma, set inwards from the gemma edge, and are also dispersed throughout the gemma body. As the plant grows, oil cells continue to be sited close to the edge of the thallus but are also dispersed throughout the thallus, including between air chambers. In this case, the identification of oil cells becomes more complicated due to their irregular distribution.

Five marker lines were identified with signals specific to oil cells. One line, ET239‐P54, was chosen as a representative oil cell line due to its specificity and strong, reliable signal (Figure 2b–f). The formation of new oil cells during thallus growth was also observable around the apical notch region, indicating the zone in which oil cell fate specification occurs (Kanazawa et al., 2020) and where the spatial dynamics controlling the distribution of oil cells happen. The insertion site in line ET239‐P54 was identified as being on chromosome 4, at site 304 117 in the MpTakv6.1 genome annotation. This location is close to two possible candidate genes [Mp4g00200 (Mapoly0162s0001) and Mp4g00180 (Mapoly0162s0003)] annotated in the *Marchantia polymorpha* v6.1 genome as belonging to the WRKY transcription factor family (WRKY12 and WRKY13 respectively), and a third possible candidate gene [Mp4g00190 (Mapoly0162s0002)] that encodes a small protein of unknown function and with no known homology to other plant genes (Figure 2g). Of these potential candidates, the WRKY12 gene Mp4g00200 is enriched within the oil cell cluster identified by scRNA‐seq (Wang et al., 2023). WRKY transcription factors are known to be involved in abiotic stress and anti‐herbivore responses (Bakshi & Oelmüller, 2014; Wani et al., 2021), and oil cells are known to have a role in deterring herbivores (Kanazawa et al., 2020). The identification and characterization of a new oil cell marker opens avenues towards producing synthetic promoters specific to these cells. This has biotechnological potential for harnessing Marchantia oil bodies for the production of specific secondary metabolites such as medicinal isoprenoids (Suire et al., 2000; Takizawa et al., 2021).

### Margin tissue

**Figure 3:**
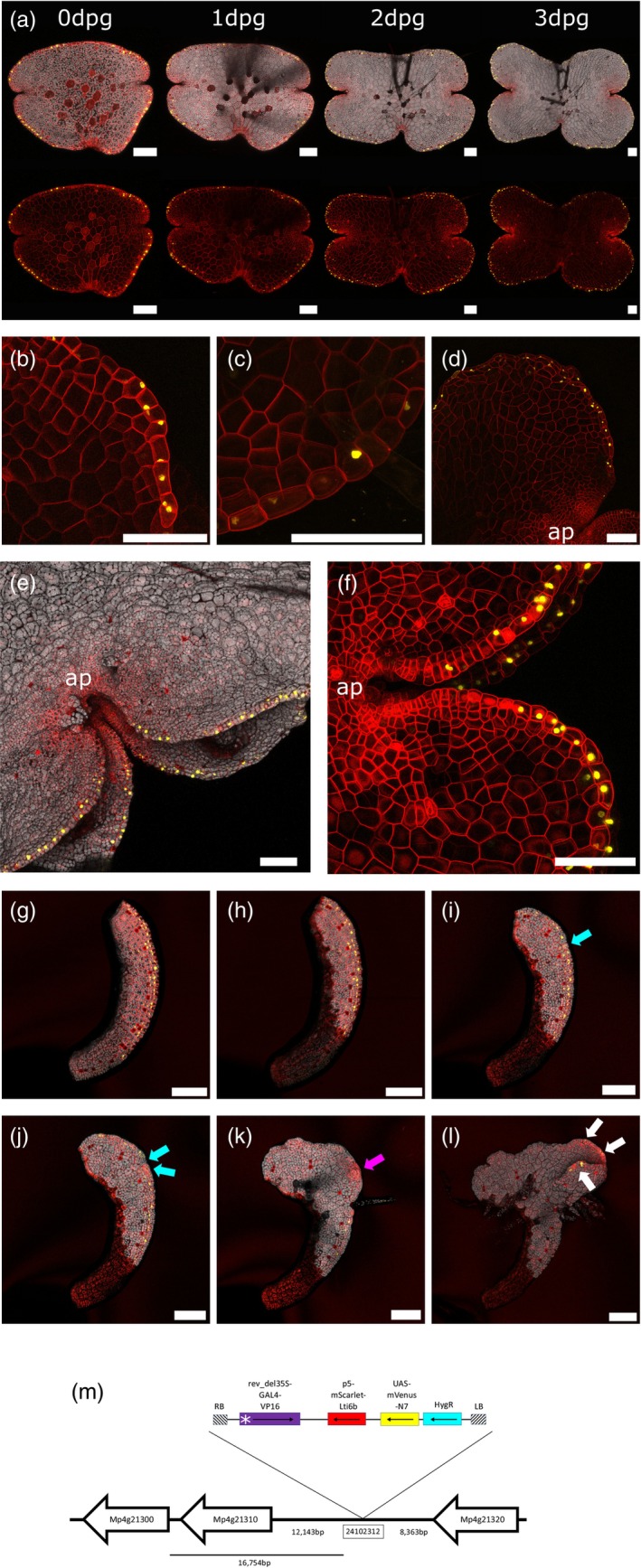
Margin tissue marker line ET239‐P64. (a) The same gemma was imaged at 0 dpg, 1 dpg, 2 dpg and 3 dpg, showing localization of the mVenus marker signal to the rows of cells running around the edge of the gemma, distal to the oil cells. The bottom image shows the same image with the chlorophyll autofluorescence channel omitted. (b) and (c) are close‐up views of margin tissue cells, showing their pillow‐shaped morphology. (d) Ventral view of a gemma imaged at 3 dpg, indicating that the marker signal is only localized to the cells at the gemma edge and that this is visible from both the dorsal and ventral views because this region is only one cell layer thick. “ap” indicates the location of the apical notch. (e) and (f) show the second row of marker signal that emerges as the gemma splits along its *z*‐axis approximately 3–5 dpg. “ap” indicates the location of the apical notch. (g–l) Isolated portion of margin imaged at 0 DaC (g), 1 DaC (h), 2 DaC (i), 3 DaC (j), 5 DaC (k) and 9 DaC (l) showing loss of margin marker signal (cyan arrows), reprogramming into new meristem (pink arrow), before appearance of new margin tissue signal de novo (white arrows). Scale bars = 100 μm. (m) is a schematic diagram illustrating the genomic location of the insert site on chromosome 4, near a putative candidate Myb transcription factor gene Mp4g21300 (Mapoly0090s0091), annotated as Mp1R‐MYB17. Neighbouring genes are indicated as white arrows, starting at the gene's start codon and pointing towards the gene's stop codon. Schematics are not drawn to scale and distances are indicated in base pairs. The T‐DNA map shows the relative positions of the minimal promoter (white asterisk), GAL4 enhancer (purple), mScarlet membrane marker (red), UAS‐mVenus nuclear marker (yellow) and hygromycin resistance gene (cyan), as well as the left and right border sequences (hatched). Black arrows point from the start codon towards the stop codon for each gene.

One of the most powerful aspects of an enhancer trap screen is its ability to mark new types of cells or tissues for further study (Shima et al., 2016; Sundaresan et al., 1995). An example of this is line ET239‐P64, which exhibits nuclear mVenus expression in the two rows of cells that run around the edge of the gemma, except at the stalk scar (Figure 3a). The signal in these marginal cells appears just beyond the apical notch, approximately 10–12 cells out from the apex cell. Here the enhancer trap line ET239‐P64 allows the characterization of these cells as having a specific “margin tissue” identity.

These cells are elongated and ‘pillow‐shaped’, are found distal to a row of oil cells, and are always found as one‐cell thick layers (Figure 3b–d). This characteristic cell morphology has been previously noted as being present at key stages in early gemma development (Kato, Yasui, & Ishizaki, 2020; Shimamura, 2016) and sporeling development (O'Hanlon, 1926). The margin tissue cells at the edge of the gemma were observed to divide both parallel and perpendicular to the gemma edge, occasionally dividing in both directions successively. While the division was usually orientated across the long axis of the cell, the direction of margin tissue cell division could not be predicted from cell shape alone, and neither cell size nor morphology was an indicator of which cells would divide. No divisions occurring in the *z*‐axis were recorded. This maintains the margin tissue as a one‐cell thick layer and as a rim of one or two cells distal to the oil cells. In common with other tissue types, cell divisions in the margins are restricted to the area surrounding the apical notch, and outside this region, margin tissue cells are mitotically inactive (see Figure S1 for examples of time course imaging with margin tissue cell divisions tracked). mVenus expression is seen stronger distal from the notch, possibly indicating a gradual cell fate transition from apical notch (i.e., meristematic) cell to margin tissue cell.

At some point after the gemma is removed from the gemma cup and growth commences, a new, additional set of margin tissue forms further in, distal from the apical notch, running parallel to the notch sides (Figure 3e,f; Movie S1 and S2). Nuclear‐localized mVenus expression indicates that the formation of this second set occurs by 5 dpg, though in some cases it was observable as early as 1 dpg. As cell division continues, and cells between the two sets of margin tissue enlarge as they move outwards from the notch region (Solly et al., 2017), a split forms in the thallus along the *z*‐axis. This *z*‐axis split marks the transition to the mature thallus form, with the appearance of air pores and air chambers in the region between the two sets of margin tissue (Apostolakos & Galatis, 1985). After this split, no new sets of margin tissues develop, and instead, the existing margin tissues grow from the apical notches. This indicates that the formation of a second set of margin tissue is an early step of the developmental sequence that creates the thicker thalloid form and that the regulation of margin tissue formation is a component of the correct development of the Marchantia thallus.

Cutting experiments were conducted using a laser ablation microscope to test whether margin tissue is specified by the simple presence of a dorso‐ventral boundary, or if margin tissue cell fate is determined by other cell associations during development. Various cuts were made by laser ablation to 0 dpg gemmae, along the gemma edge and to the apical notch region (Figure S2a–e). After observing gemma growth at regular intervals, it was consistently observed that margin tissue mVenus signal was not simply a result of there being a dorso‐ventral boundary. Only intact apical notches produced new margin tissue, as denoted by the emergence of a new mVenus signal. Margin tissue signal did not appear spontaneously at the gemma edge, but instead only appeared in cells in the region immediately outside of the apical notch and was then maintained as the gemma grew larger. Crucially, if a small portion of gemma was excised from the peripheral region, margin tissue signal was only present in those cells that were at the edge of the original intact gemma (Figure 3g,h). As meristem regeneration occurs (Kubota et al., 2013), the margin tissue signal fades [i.e., those cells lose margin tissue specification, blue arrows, Figure 3(i,j)] and the cells acquire a new fate, becoming meristematic cells. This is consistent with de‐differentiation of Marchantia cells during thallus regeneration (Nishihama et al., 2015). As the regeneration of meristematic tissues continues, a new apical notch region forms [pink arrows, Figure 3(k)]. New margin tissue is produced *de novo* from this new notch region [white arrows, Figure 3(l)]. The formation of new margin tissue occurs along the axis of the new apical notch, irrespective of other environmental cues such as gravity or light. This confirms that margin tissue is a specific tissue cell type produced from the apical notch meristem, rather than appearing purely due to external positional cues or a cell finding itself with no neighbouring cells on one side (i.e., at the edge of a gemma). This is in contrast to some cell types in the *Arabidopsis* root, where the removal of one cell type causes other cells to acquire new fates by positional cues only (Van den Berg et al., 1995), or in the leaves of angiosperms, where the presence of a dorso‐ventral boundary and adaxial‐abaxial interactions specifies leaf laminar margins (Byrne, 2012).

The margin tissue is notable in that it does not divide in the *z*‐axis, maintaining the thallus edge as one cell in thickness, and does not diversify to produce any other cell types after its initial emergence out of the apical notch. We suggest a possible model where specification or not as margin cell is the first fate choice available to cells produced by the meristematic apical cells as they leave the apical notch. If the cell is at the edge, with one side not touching any other cells, then integration of signals would allow it to detect that its position must be at the thallus edge and to adopt a margin tissue fate. If, on the other hand, the cell is in contact with other cells on all sides, then it would not acquire margin cell fate and instead follows possible “inner” specification pathways, to produce other cell types such as air chambers, oil cells, etc. An exception to this in normal development would be the formation of the second, distal set of margin tissue that emerges despite being in contact with other cells. After the second set of margin tissue emerges, no further new sets of margin tissues form (possibly due to inhibitory feedback effects) and the three‐dimensional thalloid growth of the mature gemma proceeds, unless there are other influences such as elevated auxin levels (see below). In this way, the margin tissue could play a role in defining the thalloid growth form.

The insertion site in line ET239‐P64 was identified as being on chromosome 4, at site 24 102 312 in the MpTakv6.1 genome annotation (Figure 3m). Potential candidate genes nearby are Mp4g21320 (Mapoly0090s0089, a cyclophilin‐type peptidyl‐prolyl cis‐trans isomerase), Mp4g21310 (Mapoly0090s0090, a gene with no similarity to any other genes in the Genbank/NCBI database) and a third candidate gene Mp4g21300 (Mapoly0090s0091). Mp4g21300 is annotated in the *Marchantia polymorpha* v6.1 genome as being Mp1R‐MYB17 and having significant sequence similarity to Myb transcription factors (highest similarity in Arabidopsis to Myb20 and Myb42). Mp1R‐MYB17 has been noted to be strongly up‐regulated in response to auxin (2,4‐D) treatment from RNAseq experiments (Flores‐Sandoval et al., 2018; Mutte et al., 2018). The activity of this gene has not been characterized in Marchantia, but Myb20 and Myb42 genes are known to be connected to lignification‐related processes and bark formation in vascular plants (Geng et al., 2020; Wang & Dixon, 2012) and other Myb family genes are connected to cuticle formation in Marchantia (Albert et al., 2018). While bryophytes lack lignin, they do synthesize some lignin precursors in their cuticles (Bowman et al., 2017; Kolkas et al., 2022; Renault et al., 2017). This raises the possibility that the margin tissue cell wall composition may be different from the cell walls of the rest of the thallus. Interestingly, Mp4g21300 expression is significantly enriched in one unannotated cluster (Cluster 13) of the scRNA‐seq analysis. Cluster 13 has Gene Ontology term enrichment for flavonoid and phenylpropanoid biosynthesis, as well as terms connected to cell wall biogenesis and polysaccharide biosynthesis. We speculate that Cluster 13 could be margin tissue. This would tally with Cluster 13 being a greater proportion of cells in older gemmae (31 days after germination) when the pattern of thallus growth would lead to relatively more margin tissue compared to younger, rounder gemmae (Wang et al., 2023). Combined with the auxin responsiveness of the trapped enhancer element (see below), and the possible functional role of margin tissues in Marchantia morphogenesis, this line provides a promising tool for future research.

### Margin tissue marker response to auxin treatments

The application of exogenous auxin to Marchantia thalli induces aberrant growth to produce heavy ridges (Flores‐Sandoval et al., 2015; Kato et al., 2017). To test whether the margin tissues are affected, ET239‐P64 line gemma were grown on media containing 1 μm NAA (Figure S3a,b), 3 μm NAA (Figure S3c,d) or 5 μm NAA (Figure S3e–g). Elevated auxin levels resulted in the appearance of additional mVenus signals in a dose‐dependent manner. This additional signal is not restricted to the thallus edges but is found further in towards the central zone and thallus lobes compared to untreated control plants (Figure S3h,i). Time‐lapse imaging revealed that the additional signal appears spontaneously within existing thallus cells, rather than being derived from the production of extra fluorescent cells growing out from the apical notches (Movie S3). The cells displaying nuclear‐localized mVenus do not necessarily have the characteristic “pillow‐shaped” morphology of regular margin tissue cells, even at the thallus edge where they can possess irregular protrusions (Figure S3g).

In contrast, the application of the auxin synthesis inhibitor yucasin resulted in a dramatic loss of margin tissue signal (Figure S3j,k). This may be related to the wider effects of auxin synthesis inhibitors on the morphology of the cells at the apical notch (Flores‐Sandoval et al., 2015). Gemmae grown on media supplemented with the auxin transport inhibitor TIBA did not show the altered distribution of the margin tissue signal in the young gemmae (Figure S3l,m) but did exhibit a noticeable change in disrupting the formation of the additional set of margin tissue compared to untreated control plants (Figure S3n–s). The *z*‐axis split in the gemmae failed to occur and no air chambers were formed, even in gemmae grown for up to 2 weeks (e.g. Figure S3p–s). This highlights how the formation of the second set of margin tissue is associated with the *z*‐axis split and that this is a critical stage in producing the mature thallus morphology (Shimamura, 2016).

Validation experiments were performed to verify that the approach taken in the auxin manipulation experiments did indeed affect gemmae growth and gene expression. Gemmae from line ET239‐P54 were also grown in each treatment, showing that the auxin and inhibitor treatments did not alter the enhancer trap signal non‐specifically (Figure S4a). ET239‐P54 marks oil cells, which are not known to be auxin‐responsive. Further controls used a transgenic marker line with a fluorescent protein gene driven by the promoter of the auxin synthesis gene YUCCA2 (Figure S4b). MpYUC2 marker plants demonstrated predictable responses: reduced gene expression correlated to elevated auxin levels, and increased expression under inhibitor treatment (Figure S4c). It is noteworthy that in these plants the gene expression response was experienced uniformly across the gemma, rather than being limited to any one region or tissue type. This is consistent with previous qPCR analysis, showing that auxin treatment reduces MpYUC2 expression (Mutte et al., 2018).

The effects of manipulation of auxin levels on margin marker expression strongly suggest that the associated gene expression, and the formation of margin tissue, are auxin‐dependent. This is corroborated by the spatial localization of expression of the auxin transporter gene MpPIN1 (Figure S4d). Marchantia has only one copy of this canonical polar auxin transporter. In 0 dpg gemma, PIN expression is focused around the apical notch. As the gemma grows, there is a scattered expression in the central zone, concentrated expression around the apical notch, and a chain of expression around the gemma edge. As the gemma grows further, the pattern of expression clearly shows MpPIN1 localized around the gemma edge, co‐incident with the margin tissue region. PIN gene expression is known to be up‐regulated by auxin (Paciorek et al., 2005; Tanaka et al., 2006). As with MpYUC2, the MpPIN1 marker line plants also displayed the predicted response, where elevated auxin treatment increased the distribution of MpPIN1 gene expression, particularly in the central zone of the gemma, correlated with NAA concentration (Figure S4e). In contrast, inhibitor treatment reduced MpPIN1 expression, most conspicuously around the apical notches of treated gemmae (Figure S4e). This is evidence of spatial heterogeneity of auxin in the gemma (Ishida et al., 2022).

Additionally, when over‐expression of the gene MpLAXR (MpERF20) was induced, it was observed that cells around the gemmae edges divided strongly to form cellular masses (Ishida et al., 2022). It is plausible that MpLAXR (MpERF20) is acting as part of a regulatory system for margin tissue, with high auxin levels repressing expression to specify fate. These pieces of evidence point towards auxin being a signal in the specification of cells as “outer” (i.e. margin tissue) or “inner” (i.e. not margin) immediately after they are produced from the meristematic apical notch.

### Apical notch/meristem marker lines

**Figure 4:**
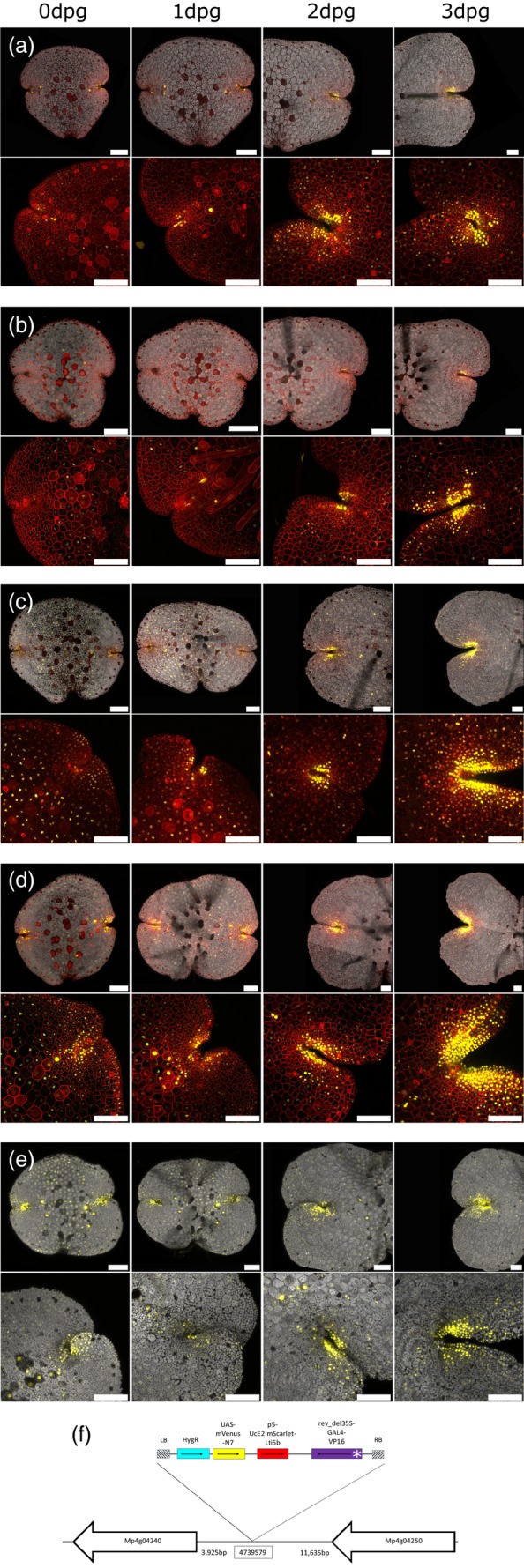
Selected marker lines for the apical notch region of the gemma. (a) ET239‐P161, (b) ET239‐P156, (d) ET239‐P49, (d) ET239‐P14 and (e) ET239‐P133. The top row in each sub‐figure is the same gemma imaged at 0 dpg, 1 dpg, 2 dpg and 3 dpg. The bottom row in each sub‐figure is a higher magnification image of a different gemma from each line imaged at 0 dpg, 1 dpg, 2 dpg and 3 dpg. Scale bars = 100 μm. Note that ET239‐P133 (e) lacks the membrane signal, possibly due to disruption of the T‐DNA insert in the genome. (f) Genomic location of the insertion site in an exemplar apical notch/meristem marker line, ET239‐P161. The location of the insert site is on chromosome 4, nearby the putative candidate gene Mp4g04240 (Mapoly0044s0049), which is annotated as containing the embryophyte‐specific domain of unknown function PTHR33782. Neighbouring genes are indicated as white arrows, starting at the gene's start codon and pointing towards the gene's stop codon. Schematics are not drawn to scale and distances are indicated in base pairs. The T‐DNA map shows the relative positions of the minimal promoter (white asterisk), GAL4 enhancer (purple), mScarlet membrane marker (red), UAS‐mVenus nuclear marker (yellow) and hygromycin resistance gene (cyan), as well as the left and right border sequences (hatched). Black arrows point from the start codon towards the stop codon for each gene.

A further benefit of enhancer trap systems is to reveal finer scale anatomy of key, but poorly understood, features. The notch‐like apical meristem of Marchantia provides a case in point. Thirteen lines were selected as having particularly strong, specific and reliable signals in and/or around the apical notch (Figure 4; Figure S5). The growth and development of the notch signals were tracked from 0 dpg to 3 dpg for each line. The distribution of signal in each line was subtly different, in some cases being restricted to the immediate apical notch [e.g. ET239‐P161 Figure 4(a), ET239‐P156 Figure 4(b)], or being found at and around the apical notch [e.g. ET239‐P49 Figure 4(c)], particularly along the flanks of the notch [e.g. ET239‐P14 Figure 4(d)]. In several cases, signals were present throughout the gemma initially, but as gemma growth proceeded it became more concentrated at the apical notch before fading as cells aged [e.g. ET239‐P133, Figure 4(e)]. It should be noted that these lines could be interpreted as marking gene expression related to meristem‐specific processes in the apical notch, or they may simply be markers of cell age and therefore represent “proxies” of meristematic activity. A further range of markers for the apical notch region is demonstrated across the eight lines shown in Figure S5(a–h).

The insertion site was identified in one line, ET239‐P161 (Figure 4a), as an exemplar of an apical notch/meristem marker line. The insertion site was located within an intergenic region of chromosome 4 (site 4 739 579 in the MpTakv6.1 genome annotation). This is adjacent to the candidate gene Mp4g04240 (Mapoly0044s0049) (Figure 4f), which has been annotated in the *Marchantia polymorpha* v6.1 genome as having a domain of unknown function PTHR33782. Analysis of scRNA‐seq data finds that expression of Mp4g04240 is enriched in the air pore cluster, although the authors do note that their analyses were limited by the fact that the proportion of cells sampled from near the notch was relatively low (Wang et al., 2023). While the molecular function of this gene and the PTHR33782 domain is unknown, BLAST searches against the EMBL/Genbank nucleotide and protein databases find that it has sequence similarity to a set of genes present in all land plants and that this gene family is unique to embryophytes. This putative candidate gene family, therefore, represents an intriguing candidate for further study in other plant groups and their meristems.

**Figure 5:**
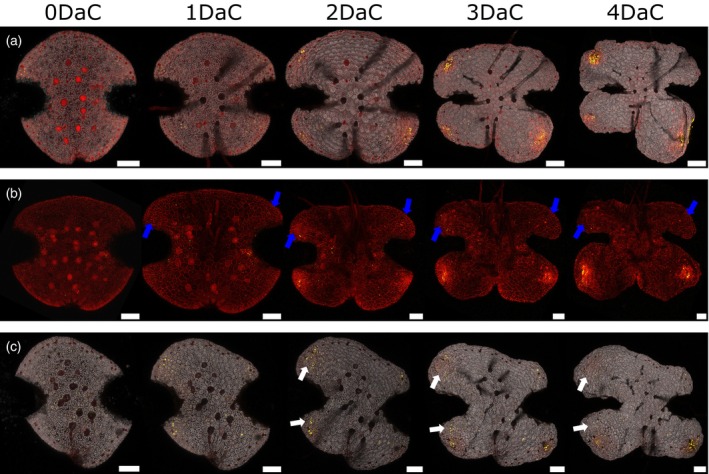
Timing of reappearance of apical notch/meristem marker signal after excision of the apical notches in selected lines. (a) Line ET239‐P161, imaged 0–4 DaC showing the reappearance of nuclear mVenus signal after 24–48 h that marks regions that regenerate full meristems by 4 DaC. (b) Line ET239‐P156 imaged 0–4 DaC, showing the reappearance of nuclear mVenus signal after 24–48 h and full meristem re‐emergence by 4 DaC. Blue arrows show regions with cell divisions early on but where no marker signal emerges and persists, showing that the marker signal is not simply related to cell division but instead is meristem‐specific. The chlorophyll autofluorescence channel has been omitted for clarity. (c) Line ET239‐P49 0–4 DaC, showing reappearance of nuclear mVenus signal after 24–48 h and full meristem re‐emergence by 4 DaC. White arrows show regions initially with cell division, but where cell division later stops and the marker signal fades. This indicates that the dynamics of the marker relate specifically to meristem regeneration. Scale bars = 100 μm.

*Marchantia polymorpha* exhibits remarkable regenerative properties, and if both apical notch regions are excised from a gemma, one or more new apical notches will regenerate and emerge from the remaining tissue, usually within 96 h (Binns & Maravolo, 1972; Kubota et al., 2013; Nishihama et al., 2015). This occurs after a burst of cell division before one or more of these proliferative patches develops into a new meristematic region and recapitulates the full apical notch morphology (Nishihama et al., 2015). We examined the apical notch marker lines by cutting away the region surrounding the apical notches in each gemma by means of laser ablation (see Methods), and then monitored gemmae daily for changes in marker signal. In all cases, the signals re‐appeared, marked the regions of regenerating meristem and persisted in newly formed apical notches. Most (10 out of 13) lines had their first new marker signal appearing 24–48 h after cutting (Figure 5a–c; Figure S6a–d,g,i,j,l). These results indicate that the lines mark processes that occur early in meristem formation/regeneration and in the acquisition of meristem fate. It is also apparent that not all patches of cell proliferation develop into fully active notches and maintain apical notch/meristem marker signals [arrows, Figure 5(b,c)]. In these cases, the marker signal is observed to fade and disappear over time, consistent with the view that these enhancer trap lines are markers of active meristematic tissue.

**Figure 6:**
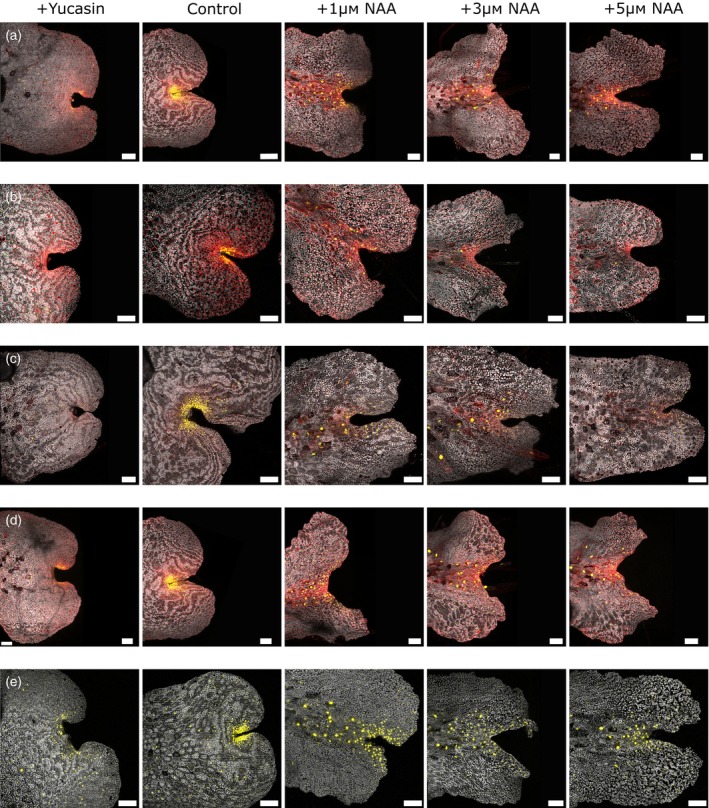
The effect of auxin manipulation of the growth media on apical notch/meristem marker line gemma in selected lines. (a) ET239‐P161 shows signal distribution changes relating to changes in notch architecture. (b) ET239‐P156 signal is reduced or eliminated under both elevated auxin and inhibitor treatments. (c) ET239‐P49 shows reduced signal under elevated auxin whereas inhibitor treatment causes changes in spatial distribution. (d) ET239‐P21 shows an elevated auxin response related to notch architecture but inhibitor treatment eliminates or strongly reduces the signal. (e) ET239‐P133 shows large spatial changes in signal distribution under both elevated auxin and inhibitor treatment, unrelated to changes in notch architecture. All gemma shown were imaged at 3 dpg. From left to right +10 μm yucasin, untreated control, +1 μm NAA, +3 μm NAA, +5 μm NAA. Scale bars = 100 μm.

The phytohormone auxin is a central player in plant meristem dynamics (Pernisová & Vernoux, 2021; Tanaka et al., 2006). Altering auxin levels in Marchantia by treatment with exogenous auxin or drugs that inhibit auxin synthesis is known to alter the apical notch region in particular (Maravolo & Voth, 1966). Therefore, we grew gemmae from each apical notch/meristem marker line supplemented with elevated auxin (1 μm, 3 μm or 5 μm NAA) or with the auxin synthesis inhibitor yucasin (10 μm) until 3 dpg, after which the plants were imaged alongside untreated controls. The results are shown in Figure 6 and Figure S7. Each line displayed changes in the spatial distribution of the nuclear signal under different auxin treatments. There were five broad classes of response: signal distribution changes relating to changes in notch architecture [e.g. ET239‐P161, Figure 6(a); Figure S7(a–d)]; reduced/no signal under both elevated auxin and inhibitor treatments [e.g. ET239‐P156, Figure 6(b); Figure S7(e–h)]; reduced signal under elevated auxin whereas inhibitor treatment only caused changes in spatial distribution [e.g. ET239‐P49, Figure 6(c); Figure S7(i,j)]; elevated auxin response relating to notch architecture but inhibitor treatment eliminating/strongly reducing signal [e.g. ET239‐P14, Figure 6(d); Figure S7(k,l)]; large spatial changes in signal distribution under both elevated auxin and inhibitor treatment, unrelated to changes in notch architecture [e.g. ET239‐P133, Figure 6(e); Figure S7(m)].

It is notable that the four lines where alteration of the auxin environment markedly reduced nuclear mVenus signal [ET239‐P75, ET239‐P82, ET239‐P127 and ET239‐P156, see Figure 6(b) and Figure S7(e–h)] all show a pattern in the control plants of having marker signal expression close to the notch. This suggests that the enhancer elements trapped in these lines and the cells and tissues marked are involved in central, core meristem functions, where perturbations of auxin levels are extremely disruptive. These were also lines where marker signal took longest to re‐appear after apical notch excision: ET239‐P75 (3 DaC), P82 (3 DaC), P127(3–4 DaC) and P156 (2 DaC, same as in uncut control plants where signal appears at 2 dpg) (see Figure 5b; Figure S6e,f,h,k). This supports the view that the genes trapped in these lines are involved in central meristem processes that only occur in mature, fully‐functioning apical notches. Further, the varied behaviour of meristem markers is evidence for proximodistal substructures in the Marchantia apical notch, which is likely more complex than previously recognized. The zonation may be more akin to the way angiosperm shoot apical meristems can be divided into a peripheral zone, central zone and organizing centre (Pernisová & Vernoux, 2021), rather than a simpler model where the Marchantia meristem region is comprised of an apical cell surrounded by merophyte cells (Shimamura, 2016; Suzuki et al., 2020).

### Using markers from enhancer trap lines to study sporeling development

Once marker lines had been characterized in the gemma, the same lines could be used to investigate tissues and cell types in other parts of the Marchantia life cycle. Spore germination and sporeling development provided a test case. While the series of morphological transitions in sporeling growth are broadly known (O'Hanlon, 1926), links with processes in gemmae growth and thallus regeneration (Nishihama et al., 2015) are unclear. While superficially similar structures are seen during development of sporelings, gemmae and mature thalli, there is scant knowledge of whether the underlying mechanisms are the same. Furthermore, it is poorly understood how important features in sporelings, such as the emergence of a thalloid growth form, are regulated (e.g. by phytohormones or photosynthesis) and how this relates to gemma growth regulation. Spores were produced by crossing wild‐type plants with a marker line from each of the categories discussed above: rhizoids (ET239‐P177), oil cells (ET239‐P54), margin tissue (ET239‐P64) and apical notch/meristem (ET239‐P161). The spores were spread on agar plates (taken as the point of germination) and individual sporelings followed on subsequent days post‐germination (dpg) in time course experiments to monitor the appearance and patterns of the nuclear mVenus signal. Here our unbiased markers provide ideal tools to interrogate and better understand sporeling development.

**Figure 7:**
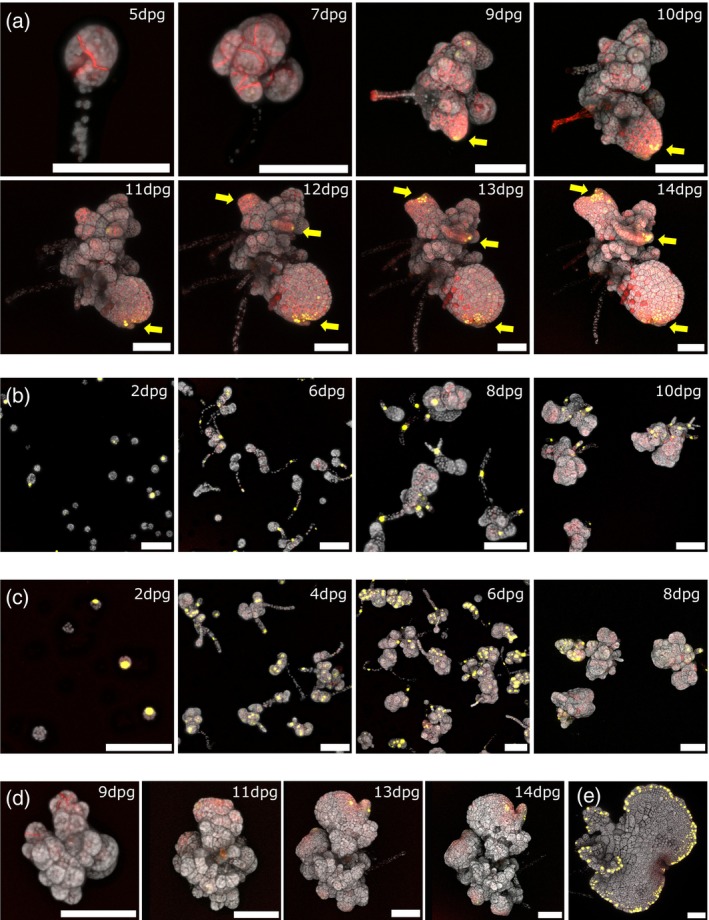
Sporeling development in enhancer trap lines. (a) Apical notch/meristem marker line ET239‐P161 sporeling developmental sequence from 5 dpg until 14 dpg. Marker signal is not simply an indicator of cell division, but its appearance only precedes the prothallus stage and marks out the notch region of a new prothallus. Multiple “pro‐notch” regions may be present in the same sporeling (yellow arrows). (b) Rhizoid marker line ET239‐P177 sporelings. mVenus marker signal appears in the rhizoids and in those cells that form rhizoids. (c) Oil cell marker line ET239‐P54 sporelings. Marker signal is present early in the germinating spores and throughout sporeling development, often in dense clusters and heterogeneously both within and between sporelings. (d) Margin tissue marker line ET239‐P64 sporeling developmental sequence, imaged from 9 dpg until 14 dpg. The mVenus signal of the margin tissue only appears after the formation of an apical notch and the emergence of the prothallus stage. (e) ET239‐P64 sporeling imaged at 12 dpg displaying strong margin tissue marker signal around the prothallus edge. Scale bars = 100 μm.

The earliest appearance of nuclear mVenus signal in sporelings from the notch/meristem marker line ET239‐P161 (Figure 7a) was in protonema at 6dpg, with most plants showing patches of marker signal by 9 —10 dpg. The appearance of the mVenus signal occurred after multiple cell divisions and the production of rhizoids in the early stages of spore germination (O'Hanlon, 1926). There was no apparent correlation between sporeling size and the timing of meristem marker appearance, nor was there any obvious morphological threshold to predict the timing or location of emergence. Protonema showed at least one patch of marker expression before proceeding to the prothallus stage, although in most cases multiple patches were present on the same protonema. Of these, one main patch proceeded to dominate and become the apical “pro‐notch” of the main emerging prothallus, although in rarer cases two patches proceeded to become growth centres and multiple prothalli would grow from the same sporeling (Figure 7a, yellow arrows). These results indicate that line ET239‐P161 is not simply a marker of cell proliferation, but specifically marks meristematic tissue that can proceed to form the apical notch of a (pro)thallus.

Further, the marker highlights a key step in sporeling development. There is a distinct difference between the protonema stage, which comprises callus‐like photosynthetic tissue and rhizoids, and the prothallus stage, where differentiated meristematic tissue has become specified in a critical developmental transition. The emergence of an organized region of meristematic tissue is crucial for producing a thalloid morphology. The ability to mark and recognize the dynamic emergence of meristematic cells in sporelings is a major advance in understanding Marchantia development. This opens up new possibilities for using gemma and sporelings to better understand the molecular and cellular processes involved in meristem regulation in Marchantia (Hirakawa et al., 2019, 2020), and plant meristems more generally (Kitagawa & Jackson, 2019; Schwartz et al., 2021).

A similar developmental sequence occurs during the regeneration of a full thallus from a small group of isolated non‐meristematic cells (Figure S8). As with spore germination, the first set of divisions proceed slowly (Figure S8a,b), and rhizoids emerge early on as the first new cell type [red arrow, Figure S8(c)]. Other than the rhizoid cells, the photosynthetic portion of the regenerating plant proceeds to a morphologically undifferentiated callus stage marked by multiple cell divisions (Figure S8d). Out of this callus one or more patches of meristematic tissue emerge [as evidenced by the appearance of ET239‐P161 marker signal, white arrows in Figure S8(e–i)]. These meristematic patches proceed to produce a “protothallus” (Figure S8j), after which one or more meristematic regions grow to dominate and become the notches of a mature thallus. The regeneration of isolated thallus cells follows a scheme outlined for protoplasts (Bopp & Vicktor, 1988; Ono et al., 1979), however this does not require chemical methods to remove the cell wall, specialized liquid media for propagation, nor an additional time interval for *de novo* synthesis of a new cell wall. Additionally, isolated cells can be identified as being derived from a specific tissue type (in this case epidermis), and the deprogramming process can be directly followed. In contrast, regeneration from protoplasts includes the uncertain loss of cell properties during isolation and unknown origin of the progenitor cells. Using this single‐cell regeneration technique, we found that cells from across all regions of a newly planted gemma, except the stalk scar, were equally capable of regenerating into new meristems. This is in contrast to regeneration studies on older thalli (10–14 dpg) which showed a bias towards regeneration from the apical edge of the ventral midrib epidermis (Ishida et al., 2022; Nishihama et al., 2015). This may be because gemmae initially lack dorsoventrality (Bowman et al., 2016; Miller & Voth, 1962), but it does suggest that cells throughout a gemma are capable of becoming adult stem cells, in contrast to, for example, Arabidopsis roots, where adult stem cells arise out the of the pericycle or pericycle‐equivalent cells (Ikeuchi et al., 2016).

Germinating ET239‐P177 spores showed marker expression in photosynthetic protonema cells before division to produce rhizoids, and in all differentiated rhizoids in the sporeling (Figure 7b). This indicates that sporeling rhizoids share some equivalence with those produced on both sides of early gemmae and ventral rhizoids of mature thalli. In contrast, observations of ET239‐P54 spores during germination and sporeling development revealed broader patterns of marker expression (Figure 7c). mVenus expression was observed in rhizoids and cells throughout the protonema and protothallus, arranged in irregular clusters throughout the sporeling. This suggests that the trapped regulatory element marked functions in wider biological processes occurring during sporeling development, not purely in oil cell formation.

Marker expression was observed in margin tissues during the growth of ET239‐P64 sporelings, consistent with observations that a distinct layer of peripheral cells is specified early in sporeling growth (O'Hanlon, 1926). No mVenus signal was observed in the early spore or protonema stages (Figure 7d). The first margin tissue marker signals were observed, after the emergence of recognizable meristematic regions (see below), generally 8—12 dpg. The morphology of the sporeling prothallus margin tissue cells is similar to those of the gemma, and the distribution of marker expression in the margin tissue signal is the same, running around the periphery of the prothallus (Figure 7e). This indicates that specification of the margin tissue is closely associated with the establishment of the thalloid form of growth.

Treatment of ET239‐P64 spores with yucasin did not prevent the appearance of margin tissue marker expression in the prothallus, nor did it affect the distribution of signal versus control sporelings (Figure S9a–d), unlike in yucasin‐treated gemma (Figure S3h–k). This indicates that the development of the prothallus and its margin tissue is different to some extent between germinating gemma and sporelings. Yucasin‐treated sporelings in this case displayed greater levels of morphological development than in the more severe phenotypes observed in sporelings from knockout mutant lines for auxin synthesis or receptor genes (Eklund et al., 2015; Suzuki et al., 2023). This suggests that, for sporelings, the yucasin treatment incompletely inhibits auxin synthesis or that sporelings are capable of intracellular detoxification of yucasin. When ET239‐P64 sporelings were grown under elevated auxin, most became stalled at the protonema stage, forming callus‐like balls of cells lacking meristematic centres. No margin tissue signal emerged in this callus‐type protonema [blue arrows, Figure S9(e–j)]. Where the sporelings did proceed to the prothallus stage in elevated auxin treatments, a margin tissue signal did appear. As in gemmae, prothalli grown under elevated auxin treatment showed additional margin marker signal in a broadly dose‐dependent manner. This demonstrates that expression of the marker for margin tissues is tightly linked to the emergence of the thalloid form and that the induction of margin tissue marker signal by auxin occurs even at the sporeling stage (Figure S9e–j).

**Figure 8:**
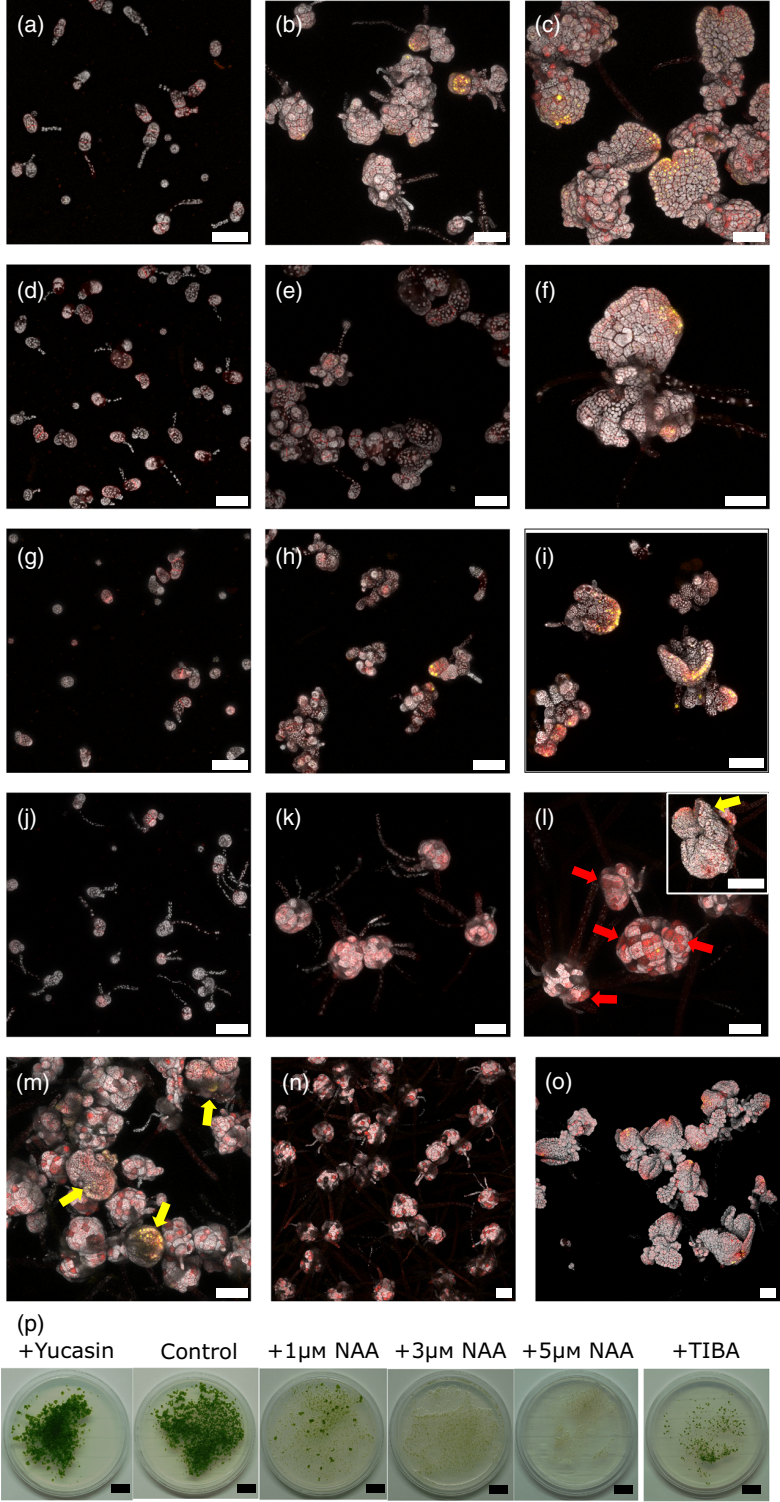
Effect of auxin manipulation on sporeling germination and development in apical notch/meristem marker plants. Control sporelings grown on untreated media imaged at (a) 4 dpg, (b) 8 dpg and (c) 12 dpg. mVenus signal marks meristematic regions. Sporelings grew on +10 μm yucasin media imaged at (d) 4 dpg, (e) 8 dpg and (f) 12 dpg. Immediately after germination and in early protonema development yucasin‐treated sporelings have enlarged vacuolated cells, however later protonema development and prothallus formation proceed as normal, with the emergence of a meristematic “pro‐notch”. Sporelings grew on +100 μm TIBA media imaged at (g) 4 dpg, (h) 8 dpg and (i) 12 dpg. Initially, sporelings have somewhat vacuolated cells and overall sporelings are slightly smaller, but there is no effect on prothallus formation and meristem emergence. Sporelings grew on +3 μm NAA media imaged at (j) 4 dpg, (k) 8 dpg and (l) 12 dpg. Early on in development, sporelings grown under elevated auxin treatment produce more rhizoids. Development then becomes stalled at the protonema stage, forming large callus‐like masses of cells that contain rhizoids, photosynthetic cells and cells containing membranous organelles (red arrows). Only occasionally do sporelings proceed to the prothallus stage, with sporelings only producing one or at most two meristem regions (inset, yellow arrow). Sporelings imaged at 12 dpg that were grown on +1 μm NAA (m), +5 μm NAA (n) or Control media (o). The number of sporelings that proceed to the prothallus stage versus those stalled at the protonema stage appears to be inversely related to the auxin concentration of the media. Where auxin‐treated sporelings progress to the prothallus stage, only one or at most two meristem regions appear per prothallus [yellow arrows (n)]. (p) Sporelings imaged at 20 dpg that were grown on: +10 μm yucasin, control, +1 μm NAA, +3 μm NAA, +5 μm NAA and + 100 μm TIBA media. Yucasin treatment produces no qualitative difference from untreated controls. TIBA treatment produces smaller sporelings. In contrast, even by 20 dpg most auxin‐treated sporelings are dead or remain at the protonema stage, with this effect greater at higher auxin concentrations. All sporelings shown were grown from ET239‐P161 spores. White scale bars = 100 μm. Black scale bars = 1 cm.

ET239‐P161 spores were also grown under different treatments to manipulate auxin levels, with media supplemented with NAA, the auxin synthesis inhibitor yucasin or the auxin transport inhibitor TIBA. This was to examine how changes in auxin levels influence meristem emergence and in particular the transition from generalized cell division in the protonema to meristem‐localized cell division in the prothallus. Compared to untreated control sporelings (Figure 8a–c), yucasin treatment initially resulted in the formation of enlarged cells after spore germination (Figure 8d,e), slowing the early development of the protonema. However, yucasin treatment did not prevent or slow the emergence of meristematic regions [as designated by the appearance of the marker signal, Figure 8(f)]. Later development to the prothallus stage was not affected, and mature sporelings were comparable to the control plants (Figure 8p). As noted for ET239‐P64 sporelings, these phenotypes are milder than is seen in knockout mutants for auxin synthesis or receptor genes (Eklund et al., 2015; Suzuki et al., 2023). TIBA treatment resulted in the appearance of elongated cells during early germination, but with less cell division so that sporelings at the protonema stage are generally smaller compared to control plants (Figure 8g,h). The cell morphology was reminiscent of yucasin‐treated protonema cells (c.f. Figure 8e,h), albeit markedly smaller. The smaller size of the protonema did not affect the emergence of the meristem signal nor the transition to the prothallus stage (Figure 8i), further supporting the finding that this developmental transition was not dependent on protonema size or morphology. The normal developmental progress of TIBA‐treated sporelings is in contrast to *Mppin1* knockout mutant sporelings (Fisher et al., 2023), where a significant proportion became stalled at the protonema stage (termed ‘blob’ stage by Fisher and colleagues). This could indicate incomplete inhibition of auxin transport in sporelings, a sporting‐specific TIBA detoxification pathway, or that MpPIN1 knockout affects sporeling development more widely than in disrupting auxin transport alone. By 20 dpg, TIBA‐treated sporelings were overall smaller than control plants but had similar morphology (Figure 8p). That inhibition of auxin synthesis and transport produces elongated or enlarged cells at the protonema stage is similar to the effects of growing sporelings under a phytochrome‐inactive Red/Far‐Red light cycle (Nishihama et al., 2015). This is reminiscent of the overlap between auxin and shade responses in other plant species (Iglesias et al., 2018).

Auxin‐treated spores showed increased rhizoid growth during the early stages of development (Figure 8j). Protonema growth continued unaffected during the early stages, however, development became stalled at this stage. The result was the production of large, ball‐shaped protonema consisting of callus‐like cells (Figure 8k), which (other than rhizoids or rhizoid precursor cells) appeared to be a poorly differentiated mass of dividing photosynthetic and cells with prominent membranous organelles [red arrows, Figure 8(l)]. Escape from this stage was possible [e.g. inset Figure 8(l)] but depended on the exogenous auxin concentration. The appearance of meristem marker signal and the transition to the prothallus stage were observed to be more frequent at 1 μm NAA (Figure 8m) versus being rare (but present) at 5 μm NAA (Figure 8n), compared to untreated control sporelings of the same age (Figure 8o). Once again, there was no link between protonema size and meristem emergence, nor were there any obvious morphological predictors for those plants that would make the transition to the prothallus stage, until the appearance of the meristem marker signal. The ‘stalling’ effect auxin has on sporeling development is still apparent at 20 dpg (Figure 8p), with sporelings at higher auxin concentrations unable to complete their development and eventually dying. The effect of auxin manipulation is in contrast to the meristematic regeneration process in gemma, where treatment with auxin synthesis or transport inhibitors causes widespread cell division but prevents the emergence of full meristems, and the application of exogenous auxin inhibits any proliferative cell division at all in cut thallus pieces (Binns & Maravolo, 1972; Ishida et al., 2022; Maravolo & Voth, 1966). Therefore, while the development of thalli from both spores and isolated cells follows the same broad set of stages, the underlying processes differ in their response to auxin manipulation treatments (Eklund et al., 2015, 2018; Fisher et al., 2023; Flores‐Sandoval et al., 2015; Ishida et al., 2022; Suzuki et al., 2023). Superficial morphological similarities but underlying mechanistic differences between regenerating thalli and sporelings have also been noted for responses to Red/Far‐Red light and sucrose supplementation (Nishihama et al., 2015). Discovering and understanding further developmental similarities and differences between these two life stages represents a promising avenue for future research.

## CONCLUSIONS

The creation of enhancer trap lines for *Marchantia polymorpha* has generated useful resources for use by the Marchantia research community. As well as the anatomical features of early gemma and spore development outlined above, the enhancer trap library contains resources for studying other tissue types, such as air pores and chambers, specific rhizoid types or the architecture of the gemma central zone. Furthermore, as the Marchantia genome has a moderate GC content of ~45% (Bowman et al., 2017; Marks et al., 2019), the distribution of insertion sites should theoretically be random (Shima et al., 2016). The current screen was far from saturating and could be easily expanded by the adoption of high throughput transformation and screening methods. This would likely reveal additional lines with novel cell markers. Targeted screening (i.e., searching for cell markers expressed specifically at certain developmental stages or growth conditions) could be repeated and applied to investigating other aspects of Marchantia development and gene expression, for example in sexual reproduction, the release of gemma dormancy or responses to abiotic stress. This could be used to probe new cell interactions and for gene identification in tissues of interest. Further, the enhancer trap scheme employs the GAL4‐VP16 gene as the primary target. This synthetic transcription factor has no natural counterpart in plant cells and acts to amplify expression of the fluorescent marker gene. GAL4‐VP16 can be used to drive ectopic expression of any other chosen gene fused to a suitable GAL4 promoter, and the pattern of expression will mirror the expression of the marker gene. Similar approaches arising from a root‐focused *Arabidopsis* enhancer trap project (Haseloff, 1999) have provided new tools and discoveries about other aspects of plant development (Laplaze et al., 2005; Radoeva et al., 2016; Wenzel et al., 2012).

The enhancer trap lines we have generated have allowed a better definition of fundamental processes in Marchantia, such as the stages of spore development. The appearance of meristematic regions was revealed to be distinct from simple localized cell division ‐ a finding facilitated by the use of an unbiased collection of meristem markers such as those generated here. We have also shown that in the spore germination process, key transitions are inhibited by applying exogenous auxin, but with smaller effects from pharmacological inhibition of auxin synthesis or transport. This contrasts with gemma regeneration and notch morphology which are heavily influenced by elevated auxin, or perturbations to auxin synthesis or transport. The enhancer trap lines will allow finer‐scale studies of notch architecture, signalling and regeneration in Marchantia, and will complement other approaches that rely on the analysis of plants at the resolution of individual cells and tissues, including single‐cell transcriptomics, promoter libraries and mutant phenotype analysis. This in turn will provide valuable insights and tools for plant science as a whole, owing to the conservation of transcription factors and genetic pathways throughout the embryophytes (Bowman et al., 2017; Eklund et al., 2018; Lu et al., 2020; Yamaoka et al., 2018)

## METHODS

### Vector construction and transformation

The enhancer trap construct was built according to the Loop assembly protocol (Pollak et al., 2019; Sauret‐Güeto et al., 2020) from L0 parts, L1 and L2 plasmids in the OpenPlant Loop assembly toolkit (Sauret‐Güeto et al., 2020). The annotated plasmid sequences of L2_GAL‐UAS_mV‐N7‐eG‐Lt‐CSA (L2_238‐CsA, p5‐35Sx2:HygR p5‐UAS:mVenus‐N7 p5UBE2:eG‐Lt del35:GAL4‐VP16) (=ET238) and L2_GAL‐UAS_mV‐N7_mS‐Lt‐CSA (L2_239‐CsA, p5‐35Sx2:HygR p5‐UAS:mVenus‐N7 p5UBE2:mS‐Lt del35:GAL4‐VP16) (=ET239) are given in Appendix S1. L2_GAL‐UAS_mV‐N7_eG‐Lt‐CSA (L2_238‐CsA, p5‐35Sx2:HygR p5‐UAS:mVenus‐N7 p5UBE2:eG‐Lt del35:GAL4‐VP16) was done by combining pCsA, L1_HygR‐Ck1, L1_UBE2:eG‐Lt‐Ck3 from the OpenPlant toolkit and new L1 plasmids L1_UAS:mV‐N7‐Ck2 and L1_del35:GAL4‐VP16_rev‐CK4. L1_UAS:mV‐N7‐Ck2 (p5‐UAS mVenus‐N7) was done with OpenPlant toolkit parts: plasmid pCK2, CD12_mVenus, CTAG_linker‐N7 and 3TERM_Nos‐35S and new synthesized L0 part PROM5_UASGAL4_min35S. L1_GAL4_rev‐CK4 (p5‐del35:GAL4‐VP16_rev‐CK4) was done with new synthesized L0 parts 3TERM_Nos_rev and PROM5CDS_del35S:GAL4‐VP16_rev and plasmid pCK4 from OpenPlant toolkit. L2_GAL‐UAS_mV‐N7_mS‐Lt‐CSA (L2_239‐CsA, p5‐35Sx2:HygR p5‐UAS:mVenus‐N7 p5UBE2:mS‐Lt del35:GAL4‐VP16) was done by combining pCsA, L1_HygR‐Ck1, L1_UAS:mV‐N7‐Ck2, L1_UBE2:mS‐Lt‐Ck3 and L1_del35:GAL4‐VP16_rev‐CK4. The minimal TATA box promoter, GAL4‐VP16 and mVenus‐N7 sequences were positioned immediately adjacent to the right border, which is first integrated into the plant genome, and provides the best opportunity for precise integration of critical elements of the enhancer trap sequence.

MpPIN and MpYUC2 marker lines were also constructed by Loop assembly to produce L2_268 35SBP:HygR2, p5‐PIN1:GAL4‐VP16, p5‐UAS‐GAL4:mVen‐N7, o941:eGFP‐Lti6b and L2_295 35S:HygR, p5‐ERF20:mVen‐N7, p5‐YUC2(5.5 kb):mTurq‐N7, p5‐o941:mScar‐Lti6b. Full annotated plasmid sequences are provided in Appendix S1. The 5′ promoter sequence for MpPIN1 started at TGAATGCGGTCGAGGACGGG (2.977 kb upstream of the start codon). The 5′ promoter sequence for MpYUC2 started at GTGGTTCCGCTCTCTCGTCG (4.97 kb upstream of the start codon).

The plasmid vectors were transformed into Marchantia sporelings (Cam accession) by Agrobacterium‐mediated transformation according to the method described in Ishizaki et al., 2016; Sauret‐Güeto et al., 2020. Transformed sporelings were grown for 2 weeks at 21°C under continuous light (intensity = 150 μmol/m^2^/sec) on 1.2% w/v agar (Melford capsules A20021) plates of Gamborg's B5 media with vitamins (Duchefa Biochemie G0210) prepared at half the manufacturer's recommended concentration and adjusted to pH 5.8. The media was supplemented with 100 μg/ml cefotaxime (BIC0111; Apollo Scientific, Bredbury, UK) to cure bacteria growth and 20 μg/ml hygromycin (10 687 010; Invitrogen [Thermo Fisher Scientific], Waltham, MA, USA) for plant selection. This media and antibiotic concentrations were used for all further growth experiments unless stated otherwise.

### Enhancer trap screening, culturing and characterization

A limited preliminary transformation and screening experiment was carried out to test the enhancer trap vector constructs before a full round of transformation and screening was performed. In the full screen, 101 transformants from the plasmid ET238 transformation were selected and isolated for further characterization. 353 transformants from the plasmid ET239 transformation were selected at random and transferred to fresh plates for further characterization. In addition, the remaining transformants were screened for mVenus fluorescence using a Leica M205 FA stereomicroscope. Fluorescing sporelings were identified, isolated and removed for further growth on fresh plates. Each sporeling was named as ET238‐PX or ET239‐PX, where PX denotes the individual plant line, numbered in order of isolation.

T0 plants were monitored for the presence and distribution of fluorescent signals as the plants grew. Gemmae were taken only from gemma cups located in regions of the thallus with signal since the clonal origin of the gemma (Kato, Yasui, & Ishizaki, 2020) avoids problems arising due to chimeric plants. Three to six gemmae from each transformant were removed from the gemma cups and grown on ½ Gamborg's media agar plates in the presence of hygromycin and cefotaxime. Gemmae were examined from 1 day after removal from the gemma cup (i.e., 1 day post‐germination, 1 dpg) until 7 dpg using a Leica M205 FA stereomicroscope equipped with filters Plant RFP (excitation filter ET560/40 nm, emission filter ET594/10 nm), ET GFP (ET470/40 nm, ET525/50 nm), ET YFP (ET500/20 nm, ET535/30 nm), ET Chlorophyll LP (ET480/40 nm, ET610 nm LP) and ET GFP LP500 (ET470/40 nm, ET500 nm LP), used, respectively, for observing mScarlet‐I, eGFP, mVenus, chlorophyll and eGFP together with chlorophyll autofluorescence (Sauret‐Güeto et al., 2020). Patterns of fluorescence (nuclear mVenus and membrane eGFP/mScarlet) were noted. Fluorescence was categorized into broad patterns according to the tissue or cell type involved. If no fluorescence was observed on any gemma by 7 dpg the line was discarded. G1 lines exhibiting gemma fluorescence were maintained on selective media by subculturing or by the propagation of gemmae and grown in a Panasonic RLR‐352 PE Climate Chamber at 16°C under continuous light at an intensity below 100 μmol/m^2^/sec. Lines were preserved as gemmae at 4°C on agar (Sauret‐Güeto et al., 2020) and preserved in long‐term frozen storage at −80°C using the CRUNC method (Takahashi & Kodama, 2020).

Plant lines with strong and consistent patterns of fluorescence that marked specific regions, tissues or cells were selected for further characterization. These lines were maintained in Aralab Fitoclima 600 growth cabinets at 21°C under constant light for faster growth. Gemmae were grown on 50 mm agar plates containing ½ Gamborg's media with hygromycin and cefotaxime for imaging using a Leica SP8 confocal microscope. Images were taken daily between 0 dpg and 4 dpg. Four to five gemmae were imaged repeatedly each day with an HC PL APO 20×/0.75 CS2 air objective; three other gemma were mounted on slides for imaging using an HC PL APO 40×/1.30 CS2 oil objective. Excitation and collection settings for each fluorophore are given in Table S1. Images were taken in photon counting mode with sequential scanning. Time gating was active to suppress autofluorescence. Maximum‐intensity projections of the images were obtained from z‐stack series, capturing z‐slices at intervals between 1–12.5 μm. Confocal microscope images are shown with the mVenus channel in yellow, mScarlet channel in red and chlorophyll autofluorescence in grey, unless otherwise noted. Stereomicroscope images were taken with a Leica M205 FA stereomicroscope equipped with a Leica DFC465 FX camera using the Plant RFP filter (excitation filter ET560/40 nm, emission filter ET594/10 nm) for the mScarlet channel, ET YFP filter (excitation filter ET500/20 nm, emission filter ET535/30 nm) for the mVenus channel and ET Chlorophyll LP filter (excitation filter ET480/40 nm, emission filter ET610 nm LP) for the chlorophyll autofluorescence channel. Images were taken with the 2×/0.10 objective at variable zoom according to the size of the gemma being imaged. Exposure time for the chlorophyll autofluorescence channel was 5 ms, 9 gain; for the mVenus channel 3000 ms, 1.7 gain; and for the mScarlet channel 100 ms, 9 gain. Gamma was set at 0.9. Z‐stack series were taken at intervals between 184 μm and 1.18 mm (for control plants) according to the size of the gemma being imaged. Extended depth‐of‐field images were created from Z‐stacks with the Leica LasX software package, using the mVenus channel as a reference. All images were processed using the Leica LasX and Fiji/ImageJ software packages. Figures were created using the Stitching (Preibisch et al., 2009) and QuickFigures (Mazo, 2021) plugins. Gemma images are presented with the stalk scar orientated towards the bottom and apical notches approximately perpendicular at each side unless otherwise noted. Excised gemma fragments and sporeling images are presented at various orientations, with apical notches marked where relevant.

### Spore production

Six lines were selected for spore production by crossing with wild‐type Cam‐1 (male) or Cam‐2 (female) plants. Plants were grown in Sac O2 Microboxes for crossing and spore production, as described in Sauret‐Güeto et al., 2020. Spores were stored at −80°C and sterilized before planting using the method described in (Sauret‐Güeto et al., 2020). Time course microscopy experiments were performed using spores germinated and grown on 50 mm agar plates containing ½ Gamborg's media with hygromycin and cefotaxime in the same growth cabinets and conditions used for gemmae growth. Spores and sporelings were imaged under the same conditions as for gemmae.

### Identification of the genomic location of insert site

gDNA was extracted from wild‐type crossed sporelings grown under hygromycin selection. Sporelings were harvested after 4.5 weeks of growth and the tissues were preserved at −80°C. gDNA extraction was performed using a modified CTAB‐based protocol (Frangedakis, 2019). Insert sites were identified using a thermal asymmetric interlaced PCR‐based method (TAIL‐PCR) (Liu et al., 1995) based on Grotewold, 2003. Primer sequences, PCR mixes and PCR protocols are given in Methods S1. PCR products were run on an agarose electrophoresis gel and the strongest bands were excised for purification using a QIAquick Gel Extraction Kit (Qiagen Ltd., Manchester, UK). The purified PCR products were sequenced directly using Sanger sequencing by Genewiz. The nucleotide sequence generated was used to search the *Marchantia polymorpha* genome v3.1 and MpTak1v6.1 using BLAST (Altschul et al., 1997) on the MarpolBase (marchantia.info) and Phytozome (phytozome‐next.jgi.doe.gov) databases. The insertion site locations identified were supported by the sequencing of multiple TAIL‐PCR products, each from different primer combinations, that all matcheo the same location in the Marchantia genome. Potential candidate genes near the insertion site were explored further using recently‐released single‐cell RNA sequencing (scRNA‐seq) data, available via wanglab.sippe.ac.cn/Marchantia‐census (Wang et al., 2023).

### Laser ablation

Plant tissues were cut by laser ablation using a Leica LMD6000 laser dissection microscope equipped with a solid‐state 355 nm cutting laser. For exciting apical notches, a 90 μm diameter circle was drawn with its centre point at the apical notch, and all tissue within this circle was ablated. The laser settings were 60 power, 45 aperture and 25 speed. All cuts were done under the 10x objective lens. Gemmae were removed from the gemma cup (i.e. 0 dpg) and planted on 50 mm agar plates. Laser ablation was performed immediately, directly on the plates, and then gemma was imaged using a Leica SP8 confocal microscope. Subsequent imaging was conducted daily from 0 days after cutting (DaC) until 4 DaC for all lines and was continued until 5 DaC in those lines where the apical notch/meristem marker had not reappeared at 4 DaC. For all lines, an uncut gemma, taken from the same gemma cup and grown on the same plate, was also imaged from 0 dpg until 4 dpg as a control. For laser ablation studies of enhancer trap line ET239‐P64, gemma was cut using the same laser settings for bulk tissue cuts under the 10x objective lens, and 60 power, 35 aperture, 20 speed under the 40× objective lens for fine‐scale cuts or ablations of a few cells at the edge of the gemma or around the apical notch. To isolate small clusters/single cells, a trace was drawn across the gemma in the pattern of radial spokes and concentric circles, and ablations done across this pattern at 60 power, 45 aperture, 30 speed under the 10× objective lens. The remaining portions of the gemma were left intact to grow and observed again at 4 DaC, upon which the largest surviving pieces of tissue were destroyed by further laser ablation, in order to provide space for the single cells/clusters of cells to grow and be accessible for imaging. This process was repeated where necessary during the time course until the single cells/clusters of cells of interest had fully regenerated into thalli.

### Auxin manipulation

For growing gemmae with elevated levels of auxin, 50 mm agar plates containing ½ Gamborg's media with hygromycin and cefotaxime were prepared with the addition of 1 μm, 3 μm or 5 μm 1‐naphthaleneacetic acid (NAA) (Sigma [Merck Life Science UK Ltd], Gillingham, UK) dissolved in 1 m NaOH and then diluted in water. Auxin synthesis inhibitor treatments were done by growing gemmae on plates made with 10 μm 5‐(4‐chlorophenyl)‐4H‐1,2,4‐triazole‐3‐thiol (yucasin) (ChemCruz, Santa Cruz Biotechnology, Heidelberg, Germany) or 100 μm L‐kynurenine (Sigma) dissolved in DMSO (Tsugafune et al., 2017). Auxin transporter inhibitor treatments were done by growing gemmae on plates made with 100 μm 2,3,5‐triiodobenzoic acid (TIBA) (Aldrich [Merck Life Science UK Ltd]) dissolved in water. Media plates were stored at 4°C wrapped in foil to protect from light and were used within 6 weeks of preparation. Auxin treatment experiments were all conducted with plants grown in the same growth cabinets as the control plants, with 4–7 gemmae grown under each treatment.

## CONFLICTS OF INTEREST

The authors declare that there are no conflicts of interests for this work.

## Supporting information


**Movie S1.** Time lapse video of the margin marker line ET239‐P64 illustrating formation of a second row of margin tissue and *z*‐axis split of the thallus. Initially margin tissue signal is only present around the thallus edge. After 12 h, the first signal appears from the second, inner row of new margin tissue at the apical notch at the left of the video. By 24 h elapsed this second row has a prominent signal and by day 2 the *z*‐axis split is clearly visible in the left apical notch. Gemma imaging began immediately after removal from the gemma cup (0 dpg), and the time elapsed is shown at the top. The mScarlet channel has been removed for clarity. Scale bar = 100 μm.

**Movie S2.** Time lapse video of the margin marker line ET239‐P64 illustrating the formation of a second row of margin tissue and *z*‐axis split of the thallus. Initially, the margin tissue signal is only present around the thallus edge. After 38.52 h, the first signal appears from the second, inner row of new margin tissue at the apical notch on the left of the video and appears after 43.656 h on the apical notch on the right. By the end of the time‐lapse (64.2 h elapsed), this second row has a prominent signal, and the *z*‐axis split is beginning in the apical notch on the left. Comparison with Movie S1 illustrates the variability between plants in the timing of this developmental stage, but that the formation of the second row of margin tissue precedes the z‐axis split, which in turn precedes the formation of the mature, fully three‐dimensional thallus with air pores and air chambers. Gemma imaging began immediately after removal from the gemma cup (0 dpg), and the time elapsed is shown at the top. Scale bar = 100 μm.

**Movie S3.** Time lapse video of the margin marker line ET239‐P64 grown on media supplemented with + 1 μm NAA, showing the spontaneous formation of margin tissue marker signals deep in the thallus. Abundant marker signal is present in the thallus, away from the edges and clearly inside the row of oil cells that is inside the margin tissue in normal development. New nuclear mVenus signal appears at random throughout the video in the thallus. This is not derived by the production of new margin tissue from the apical notch (cf. Movie S1 and S2). There is no evidence (such as new cell membrane formation, binary fission or reconstitution of fluorescent nuclei) that the new cells with marker signal are derived from existing margin tissue or thallus cells with marker signal. Instead, the gene enhancer elements trapped are being spontaneously turned on, linked to the elevated auxin treatment. Gemmae imaging began at 1 dpg, and elapsed time is shown at the top. The chlorophyll autofluorescence channel has been omitted for clarity. Scale bar = 100 μm.

**Figure S1.** Tracking cell division in the margin tissue cells located at the edge of the gemma. The margin tissue cells located right at the gemma edge were monitored for division during a time course with daily confocal imaging. Cell division events were identified by the formation of new cell membranes, as shown by the mScarlet marker, since the previous image. The tracking finds that these cells can divide both parallel to the gemma edge (i.e. transverse, denoted by plus, +) and perpendicular to the gemma edge (i.e. anticlinal, denoted by circle, o), but not in the z axis (i.e. longitudinally). Cells can undergo transverse division after anticlinal division (denoted by asterisk, *) and vice versa (denoted by circled plus ⊕), or repeating anticlinal division (bold circles o). Most cell division in the margin tissue occurs around the apical notch (ap), with margin tissue cells further away from the notch being mitotically inactive. Cells are marked with a symbol when they have divided in the next time frame, with mother and daughter cells sharing the same colour symbols (note asterisks, denoting anticlinal followed by transverse division, are put outside the mother cell for clarity). (a) imaged 0 dpg (left) and 1 dpg (right). (b) imaged 0 dpg (left) and 1 dpg (right). (c) imaged 0 dpg (left) and 1 dpg (right). (d) imaged 0 DaC (left) and 1 DaC (cutting done on 0 dpg gemma). (e) imaged 1 dpg (left) and 2 dpg (right). (f) imaged 2 dpg (left) and 3 dpg (right). (g) imaged 0–2 dpg. (h) imaged 0–2 dpg. (i) imaged 1–3 dpg. (j,k,l) imaged 0–3 dpg. (m) imaged 0–3 DaC (cutting done on 0 dpg gemma). All gemma shown are from the margin tissue marker line ET239‐P64. The chlorophyll autofluorescence channel has been omitted for clarity in all images. Scale bars = 50 μm.
**Figure S2.** Laser ablation cutting and ventral view demonstrates that line ET239‐P64 marks the cells that make up the margin tissue. (a) Gemma with all edges removed. (b) Gemma with small portion of edge removed (marked by green arrows) (c) Gemma with both notches removed but all edges intact (e) Gemma with one notch removed, other notch and all edges intact (e) Isolated gemma notch. Scale bars = 100 μm.
**Figure S3.** The effect of auxin manipulation of growth media on the margin tissue marker in line ET239‐P64. (a,b) +1 μm NAA treated gemmae at 3 dpg. (c,d) +3 μm NAA treated gemmae at 3 dpg. (e–g) +5 μm NAA treated gemmae at 3 dpg. (g) is a close up image of a +5 μm NAA treated plant showing cells exhibiting marker signal deeper inside the thallus and with irregular protrusions. (h,i) Untreated control gemmae at 3 dpg. (j,k) +10 μm yucasin treated gemmae at 3 dpg. (l,m) +100 μm TIBA treated gemmae at 6 dpg. Time course of gemma either untreated control (n,o) or treated with 100 μm TIBA (p–s) imaged by Leica M205 FA stereomicroscope. mVenus channel is shown in yellow, mScarlet channel in magenta and chlorophyll autofluorescence chanel in grey. From left to right: 1 dpg, 2 dpg, 3 dpg, 4 dpg, 5 dpg, 7 dpg, 9 dpg, 9 dpg (close up of notch of 9 dpg plant shown in (q) and (s), ap marking the apical notch). The first row of margin tissue is intact in both conditions, as shown by the marker signal being present. However, there is no formation of the second row of margin tissue, no *z*‐axis split and no air chambers form even 7 days after removal from the gemma cup, in comparison to the normal development of the untreated control plants. Scale bars = 100 μm.
**Figure S4.** Effect of auxin manipulation on ET239‐P54 (oil cell), MpYUC2 and MpPIN1 marker lines. (a) ET239‐P54 marker line gemmae at 3 dpg grown on media supplemented with +10 μm yucasin, untreated control, +1 μm NAA, +3 μm NAA or +5 μm NAA. Auxin manipulation does not appreciably change oil cell formation nor marker signal expression. (b) Characterization of MpYUC2 marker line. The top row is the same gemma imaged at 0 dpg, 1 dpg, 2 dpg, 3 dpg. mTurquoise channel shown in yellow. The bottom row is a higher magnification image of the area around the apical notch of different gemmae at 0 dpg, 1 dpg, 2 dpg, 3 dpg. mTurquoise channel shown in yellow. (c) MpYUC2 marker line gemmae at 3 dpg grown on media supplemented with +10 μm yucasin, untreated control, +1 μm NAA, +3 μm NAA or +5 μm NAA. Inhibiting auxin synthesis strongly up‐regulates MpYUC2 expression, whereas application of exogenous auxin decreases MpYUC2 expression. The effects are observed all across the gemma. mTurquoise channel shown in yellow. (d) Characterization of MpPIN1 marker line. The top row is the same gemma imaged at 0 dpg, 1 dpg, 2 dpg, 3 dpg. The bottom row is a higher magnification image of the area around the apical notch of different gemmae at 0 dpg, 1 dpg, 2 dpg, 3 dpg. These images show the prominent MpPIN1 expression in the margin tissue region, particularly illustrating the formation of the second row of margin tissue at 3 dpg. (e) MpPIN1 marker line gemmae at 3 dpg grown on media supplemented with +10 μm yucassin, untreated control, +1 μm NAA, +3 μm NAA or +5 μm NAA. Inhibiting auxin synthesis causes lower MpPIN1 expression, whereas application of exogenous auxin strongly increases MpPIN1 expression, particularly in the central zone of the gemmae. Scale bars = 100 μm.
**Figure S5.** Apical notch/meristem marker lines. (a) ET238‐P25 (b) ET239‐P21, (c) ET239‐P33, (d) ET239‐P75, (e) ET239‐P82, (f) ET239‐P125, (g) ET239‐P127, (h) ET239‐P153. Top row in each sub‐figure is the same gemma, imaged at 0 dpg, 1 dpg, 2 dpg, 3 dpg. Bottom row in each sub‐figure is higher magnification image of different gemmae from each line, imaged at 0 dpg, 1 dpg, 2 dpg, 3 dpg. Scale bars = 100 μm.
**Figure S6.** Timing of the reappearance of apical notch/meristem marker signal after excision of the apical notches. (a) ET238‐P25 imaged from 0 DaC until 4 DaC. (b) ET239‐P14 imaged from 0 DaC until 4DaC. (c) ET239‐P21 imaged from 0DaC until 4DaC. (d) ET239‐P33 imaged from 0DaC until 4DaC. (e) ET239‐P75 imaged from 0DaC until 5DaC. (f) ET239‐P82 imaged from 0DaC until 4DaC. (g) ET239‐P125 imaged from 0DaC until 4DaC. (h) ET239‐P127 imaged from 0DaC until 5DaC. (i) ET239‐P133 imaged from 0DaC until 4DaC. (j) ET239‐P153 imaged from 0DaC until 4DaC. (k) ET239‐P156 imaged from 0DaC until 4DaC [chlorophyll autofluorescence channel included, cf. Figure 5(b)] (l) ET239‐P161 imaged from 0DaC until 4DaC [chlorophyll autofluorescence channel omitted, cf. Figure 5(a)]. Scale bars= 100 μm.
**Figure S7.** Auxin manipulation treatments of apical notch/meristem marker line gemmae. (a) ET239‐P161 whole gemma images in top row, with additional gemmae shown in the row below without chlorophyll autofluorescence. (b) ET239‐P33; with additional gemmae shown in the row below without chlorophyll autofluorescence. (c) ET239‐P153; with additional gemmae shown in the row below without chlorophyll autofluorescence. (d) ET239‐P125; whole gemma images of a gemmae in the top and middle rows; the chlorophyll autofluorescence channel was omitted from images in the bottom row. The lines shown in (a–d) show signal distribution changes in respone to auxin manipulation that relates to changes in notch architecture. (e) ET239‐P156 with chlorophyll autofluorescence channel omitted. (f) ET239‐P75; with chlorophyll autofluorescence channel omitted in images of different gemmae in bottom row. (g) ET239‐P82. (h) ET239‐P127. The lines shown in (e–h) have signal that is dramatically reduced or eliminated under elevated auxin treatments and auxin synthesis inhibitor treatment. (i) ET239‐P49 with chlorophyll autofluorescence channel omitted. (j) ET238‐P25. The lines shown in (i,j) have reduced signal under elevated auxin treatments, whereas auxin synthesis inhibitor treatment causes changes in spatial distribution of signals. (k) ET239‐P21 with chlorophyll autofluorescence channel omitted. (l) ET239‐P14; with chlorophyll autofluorescence channel omitted in images of a gemmae in bottom row. The lines shown in (k,l) has a response to elevated auxin treatments that relates to notch architecture, but auxin synthesis inhibitor treatment eliminates or markedly reduces signal. (m) ET239‐P133 whole gemma images. This line shows large spatial changes in signal distribution under both elevated auxin and inhibitor treatment, that appears to be unrelated to changes in notch architecture. All gemma shown were imaged at 3 dpg. In all sub‐figures from left to right: +10 μm yucasin, untreated control, +1 μm NAA, +3 μm NAA, +5 μm NAA. Scale bars = 100 μm.
**Figure S8.** Developmental sequence of thallus regeneration from a small cluster of isolated gemma cells. Cells were isolated from the periphery of a 0DaC gemma from the apical notch/meristem marker line ET239‐P161 by laser ablation. Images of the cells shown were taken daily from 5DaC (a) until 14DaC (j). Initially cell division proceeds with no marker signal apparent, to form a callus‐like mass of photosynthetic cells with occasional rhizoids (red arrows). Marker signal appearance precedes and marks out the regenerating meristem region (white arrows) that will go on to form the apical notch of a new thallus. Scale bars = 50 μm.
**Figure S9.** Effect of auxin manipulation on spore germination and development in margin tissue marker (ET239‐P64) sporelings. (a,b) Sporelings imaged 12 dpg at the prothallus stage grown on control media. (c,d) Sporelings imaged 12 dpg at the prothallus stage grown on +10 μm yucasin media. These show normal appearance of the prothallus and margin tissue marker signal. Sporelings imaged at 14 dpg at the prothallus stage grown on +1 μm NAA (e,f), +3 μm NAA (g,h) and +5 μm NAA (i,j) media. Elevated auxin treatment retards sporeling development with prothalli taking longer to emerge, hence this stage not being observed until 14 dpg. Even with the extended growth time most sporelings grown under elevated auxin did not proceed to the prothallus stage at all. Instead, these sporelings formed large callus‐type protonema (blue arrows) and did not produce any margin tissue nor exhibit any margin tissue marker signal. Those sporelings that did proceed to the prothallus stage displayed elevated levels of margin tissue marker signal, correlated with the exogenous auxin concentration. This is similar to the situation in ET239‐P64 gemma grown under elevated auxin levels (see Figure S3a–g). Scale bars = 100 μm.

**Appendix S1.** Annotated sequence description of the plasmids used for the enhancer trap experiments L2‐238‐CsA (16,040 bp), L2_239‐CsA (16,019 bp), L2_295‐CsA (23,002 bp), L2_268‐CsA (19,174 bp). The sequences and annotation are provided in Genbank format.

**Methods S1.** TAIL‐PCR protocol for detection of enhancer trap insertion sites in the *Marchantia polymorpha* genome.

**Table S1.** Excitation and collection wavelengths used in confocal microscope imaging.
