# Supplementary material for: An enhancer trap system to track developmental dynamics in Marchantia polymorpha
Source: Plant J. 2023 Aug 15;116(2):604–28. doi: 10.1111/tpj.16394 (PMC10952768; doi:10.1111/tpj.16394)
Supplement: Supplementary file 4 — Figure S1. Tracking cell division in the margin tissue cells located at the edge of the gemma. The margin tissue cells located right at the gemma edge were monitored for division during a time course with daily confocal imaging. Cell division events were identified by the formation of new cell membranes, as shown by the mScarlet marker, since the previous image. The tracking finds that these cells can divide both parallel to the gemma edge (i.e. transverse, denoted by plus, +) and perpendicular to the gemma edge (i.e. anticlinal, denoted by circle, o), but not in the z axis (i.e. longitudinally). Cells can undergo transverse division after anticlinal division (denoted by asterisk, *) and vice versa (denoted by circled plus ⊕), or repeating anticlinal division (bold circles o). Most cell division in the margin tissue occurs around the apical notch (ap), with margin tissue cells further away from the notch being mitotically inactive. Cells are marked with a symbol when they have divided in the next time frame, with mother and daughter cells sharing the same colour symbols (note asterisks, denoting anticlinal followed by transverse division, are put outside the mother cell for clarity). (a) imaged 0 dpg (left) and 1 dpg (right). (b) imaged 0 dpg (left) and 1 dpg (right). (c) imaged 0 dpg (left) and 1 dpg (right). (d) imaged 0 DaC (left) and 1 DaC (cutting done on 0 dpg gemma). (e) imaged 1 dpg (left) and 2 dpg (right). (f) imaged 2 dpg (left) and 3 dpg (right). (g) imaged 0–2 dpg. (h) imaged 0–2 dpg. (i) imaged 1–3 dpg. (j,k,l) imaged 0–3 dpg. (m) imaged 0–3 DaC (cutting done on 0 dpg gemma). All gemma shown are from the margin tissue marker line ET239‐P64. The chlorophyll autofluorescence channel has been omitted for clarity in all images. Scale bars = 50 μm. Figure S2. Laser ablation cutting and ventral view demonstrates that line ET239‐P64 marks the cells that make up the margin tissue. (a) Gemma with all edges removed. (b) Gemma with small portion of edge removed ( [file TPJ-116-604-s004.pdf]

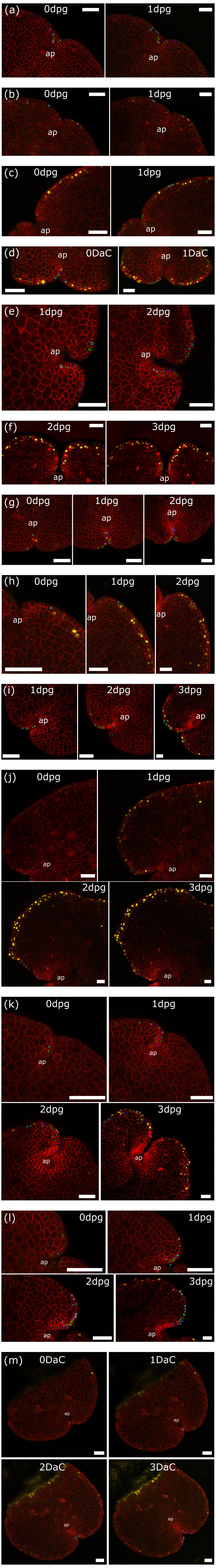

Figure S1. Tracking cell division in the margin tissue cells located at the edge of the gemma. The margin tissue cells located right at the gemma edge were monitored for division during a time course with daily confocal imaging. Cell division events were identified by the formation of new cell membranes, as shown by the mScarlet marker, since the previous image. The tracking finds that these cells can divide both parallel to the gemma edge (i.e. transverse, denoted by plus, +) and perpendicular to the gemma edge (i.e. anticlinal, denoted by circle, o), but not in the z axis (i.e. longitudinally). Cells can undergo transverse division after anticlinal division (denoted by asterisk, *) and vice versa (denoted by circled plus ⊕), or repeating anticlinal division (bold circles **o**). Most cell division in the margin tissue occurs around the apical notch (ap), with margin tissue cells further away from the notch being mitotically inactive. Cells are marked with a symbol when they have divided in the next time frame, with mother and daughter cells sharing the same colour symbols (note asterisks, denoting anticlinal followed by transverse division, are put outside the mother cell for clarity). (a) imaged 0dpg (left) and 1dpg (right). (b) imaged 0dpg (left) and 1dpg (right). (c) imaged 0dpg (left) and 1dpg (right). (d) imaged 0DaC (left) and 1DaC (cutting done on 0dpg gemma). (e) imaged 1dpg (left) and 2dpg (right). (f) imaged 2dpg (left) and 3dpg (right). (g) imaged 0-2dpg. (h) imaged 0-2dpg. (i) imaged 1-3dpg. (j), (k) and (l) imaged 0-3dpg. (m) imaged 0-3 DaC (cutting done on 0dpg gemma). All gemma shown are from the margin tissue marker line ET239-P64. The chlorophyll autofluorescence channel has been omitted for clarity in all images. Scale bars= 50μm.

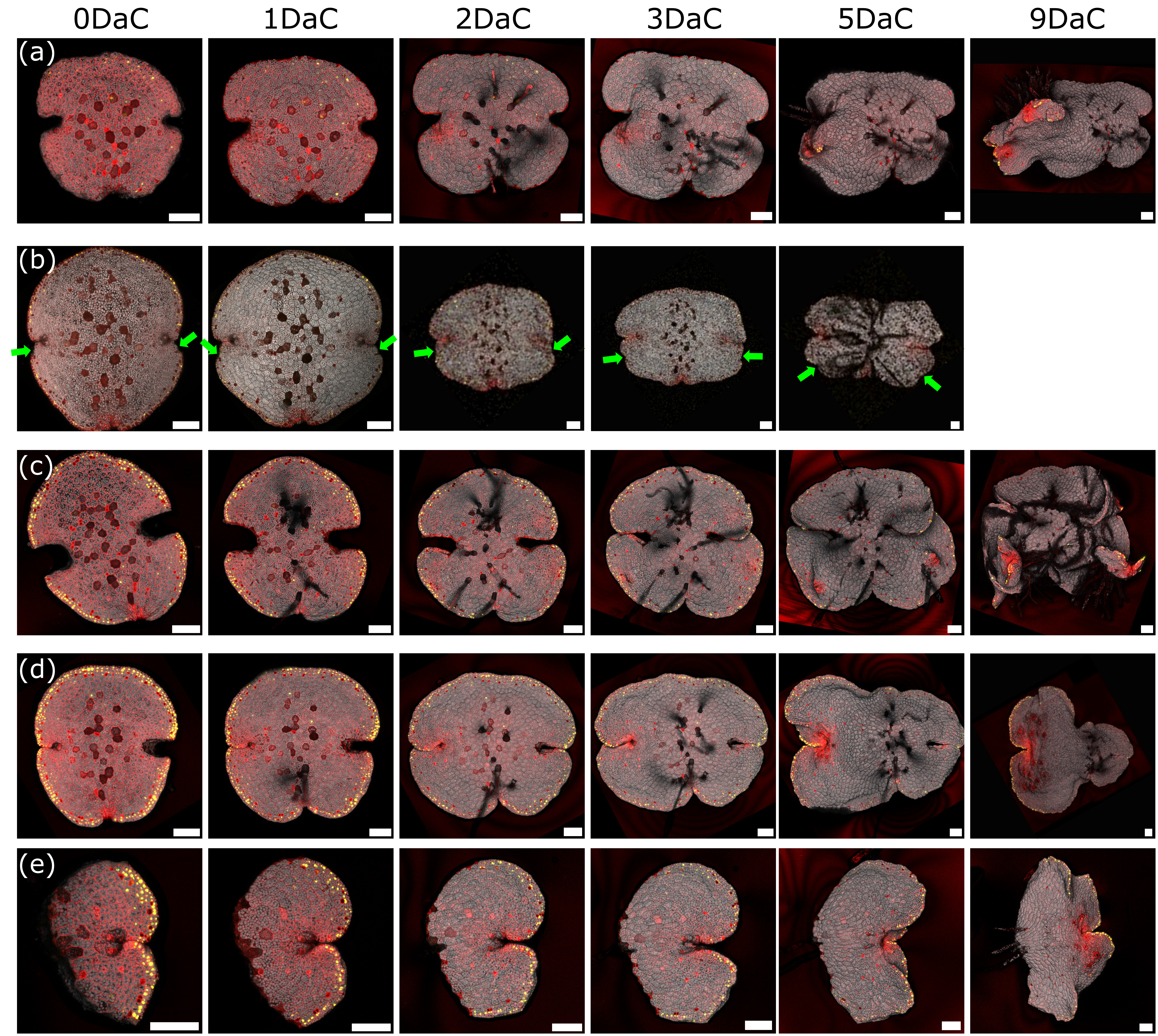

Figure S2. Laser ablation cutting and ventral view demonstrates that line ET239-P64 marks the cells that make up the margin tissue. (a) Gemma with all edges removed. (b) Gemma with small portion of edge removed (marked by green arrows) (c) Gemma with both notches removed but all edges intact (e) Gemma with one notch removed, other notch and all edges intact (e) Isolated gemma notch. Scale bars= 100µm.

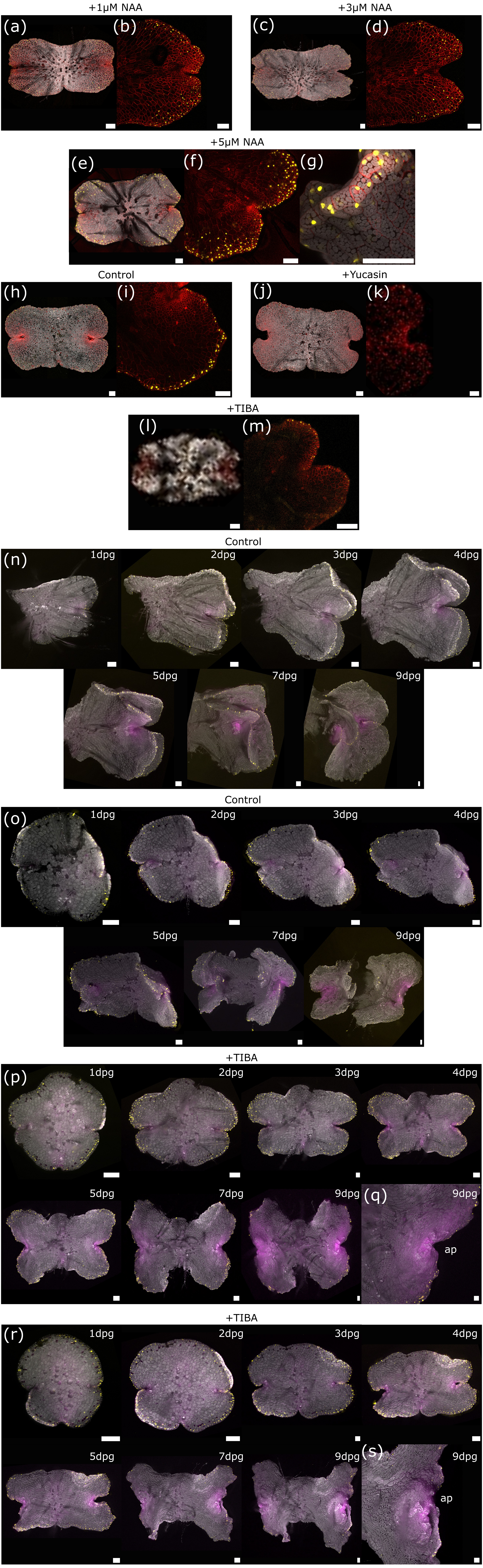

Figure S3. The effect of auxin manipulation of growth media on the margin tissue marker in line ET239-P64. (a), (b) +1μM NAA treated gemmae at 3dpg. (c), (d) +3μM NAA treated gemmae at 3dpg. (e), (f), (g) +5μM NAA treated gemmae at 3dpg. (g) is a close up image of a +5μM NAA treated plant showing cells exhibiting marker signal deeper inside the thallus and with irregular protrusions. (h), (i) Untreated control gemmae at 3dpg. (j), (k) +10μM yucasin treated gemmae at 3dpg. (l), (m) +100μM TIBA treated gemmae at 6dpg. Time course of gemma either untreated control ((n), (o)) or treated with 100μM TIBA ((p), (q), (r), (s)) imaged by Leica M205 FA stereomicroscope. mVenus channel is shown in yellow, mScarlet channel in magenta and chlorophyll autofluorescence chanel in grey. From left to right: 1dpg, 2dpg, 3dpg, 4dpg, 5dpg, 7dpg, 9dpg, 9dpg (close up of notch of 9dpg plant shown in (q) and (s), ap marking the apical notch). The first row of margin tissue is intact in both conditions, as shown by the marker signal being present. However, there is no formation of the second row of margin tissue, no z-axis split and no air chambers form even 7 days after removal from the gemma cup, in comparison to the normal development of the untreated control plants. Scale bars= 100μm.

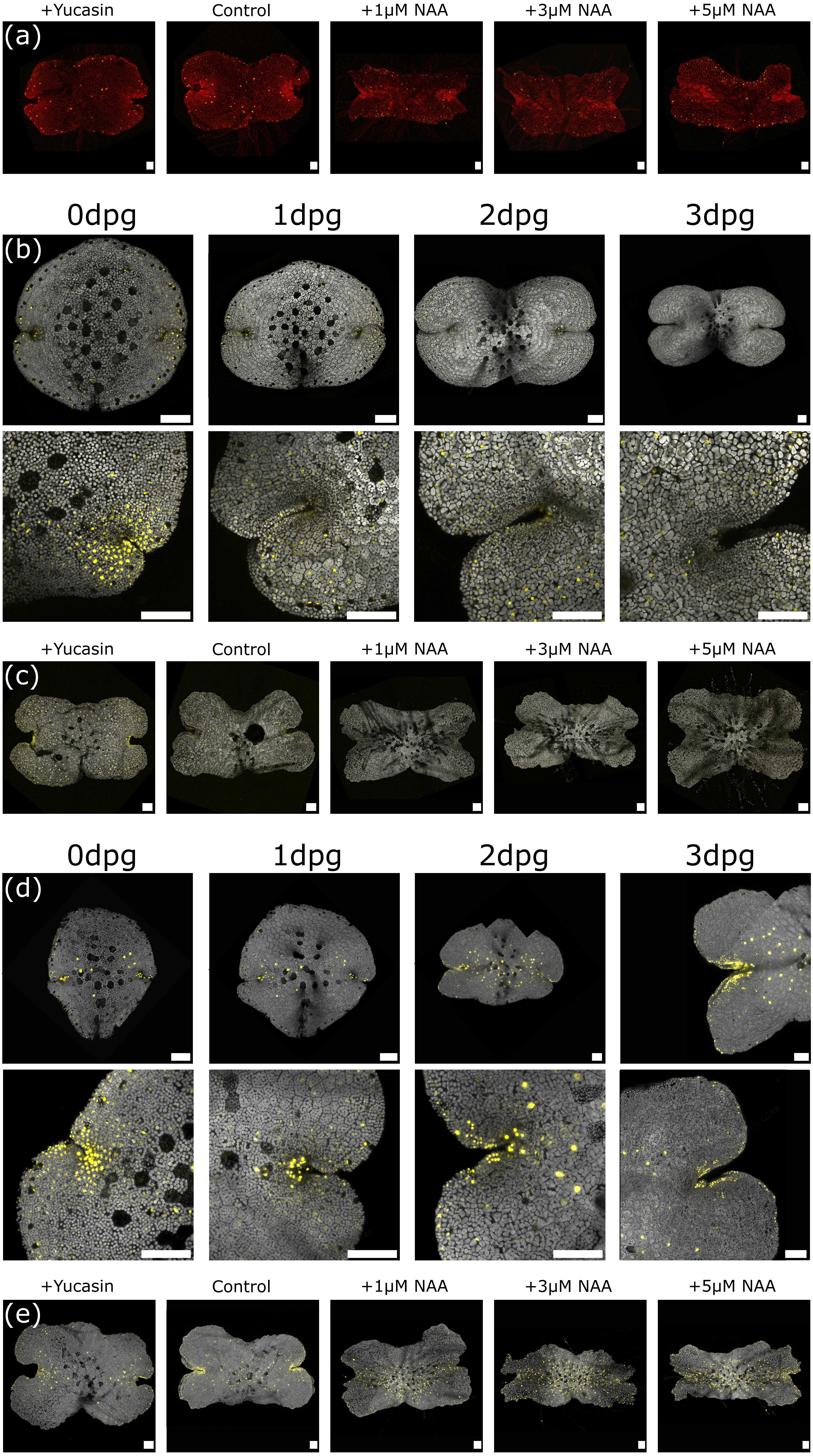

Figure S4 Effect of auxin manipulation on ET239-P54 (oil cell), MpYUC2 and MpPIN1 marker lines. (a) ET239-P54 marker line gemmae at 3dpg grown on media supplemented with +10μM yucasin, untreated control, +1μM NAA, +3μM NAA or +5μM NAA. Auxin manipulation does not appreciably change oil cell formation nor marker signal expression. (b) Characterization of MpYUC2 marker line. The top row is the same gemma imaged at 0dpg, 1dpg, 2dpg, 3dpg. mTurquoise channel shown in yellow. The bottom row is a higher magnification image of the area around the apical notch of different gemmae at 0dpg, 1dpg, 2dpg, 3dpg. mTurquoise channel shown in yellow. (c) MpYUC2 marker line gemmae at 3dpg grown on media supplemented with +10μM yucasin, untreated control, +1μM NAA, +3μM NAA or +5μM NAA. Inhibiting auxin synthesis strongly up-regulates MpYUC2 expression, whereas application of exogenous auxin decreases MpYUC2 expression. The effects are observed all across the gemma. mTurquoise channel shown in yellow. (d) Characterization of MpPIN1 marker line. The top row is the same gemma imaged at 0dpg, 1dpg, 2dpg, 3dpg. The bottom row is a higher magnification image of the area around the apical notch of different gemmae at 0dpg, 1dpg, 2dpg, 3dpg. These images show the prominent MpPIN1 expression in the margin tissue region, particularly illustrating the formation of the second row of margin tissue at 3dpg. (e) MpPIN1 marker line gemmae at 3dpg grown on media supplemented with +10μM yucassin, untreated control, +1μM NAA, +3μM NAA or +5μM NAA. Inhibiting auxin synthesis causes lower MpPIN1 expression, whereas application of exogenous auxin strongly increases MpPIN1 expression, particularly in the central zone of the gemmae. Scale bars =100μm.

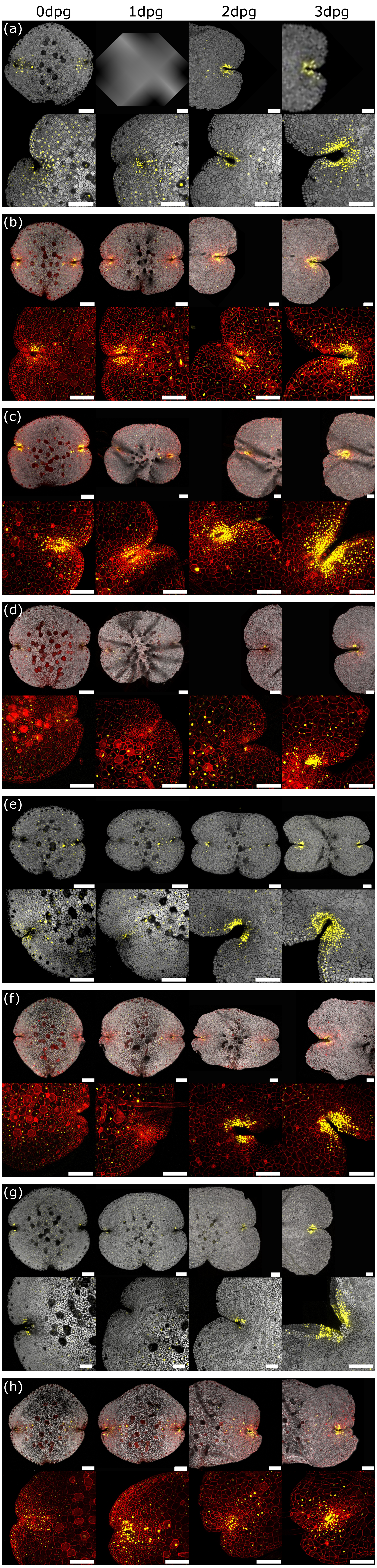

Figure S5 Apical notch/meristem marker lines. (a) ET238-P25 (b) ET239-P21, (c) ET239-P33, (d) ET239-P75, (e) ET239-P82, (f) ET239-P125, (g) ET239-P127, (h) ET239-P153. Top row in each sub-figure is the same gemma, imaged at 0dpg, 1dpg, 2dpg, 3dpg. Bottom row in each sub-figure is higher magnification image of different gemmae from each line, imaged at 0dpg,1dpg, 2dpg, 3dpg. Scale bars= 100µm.

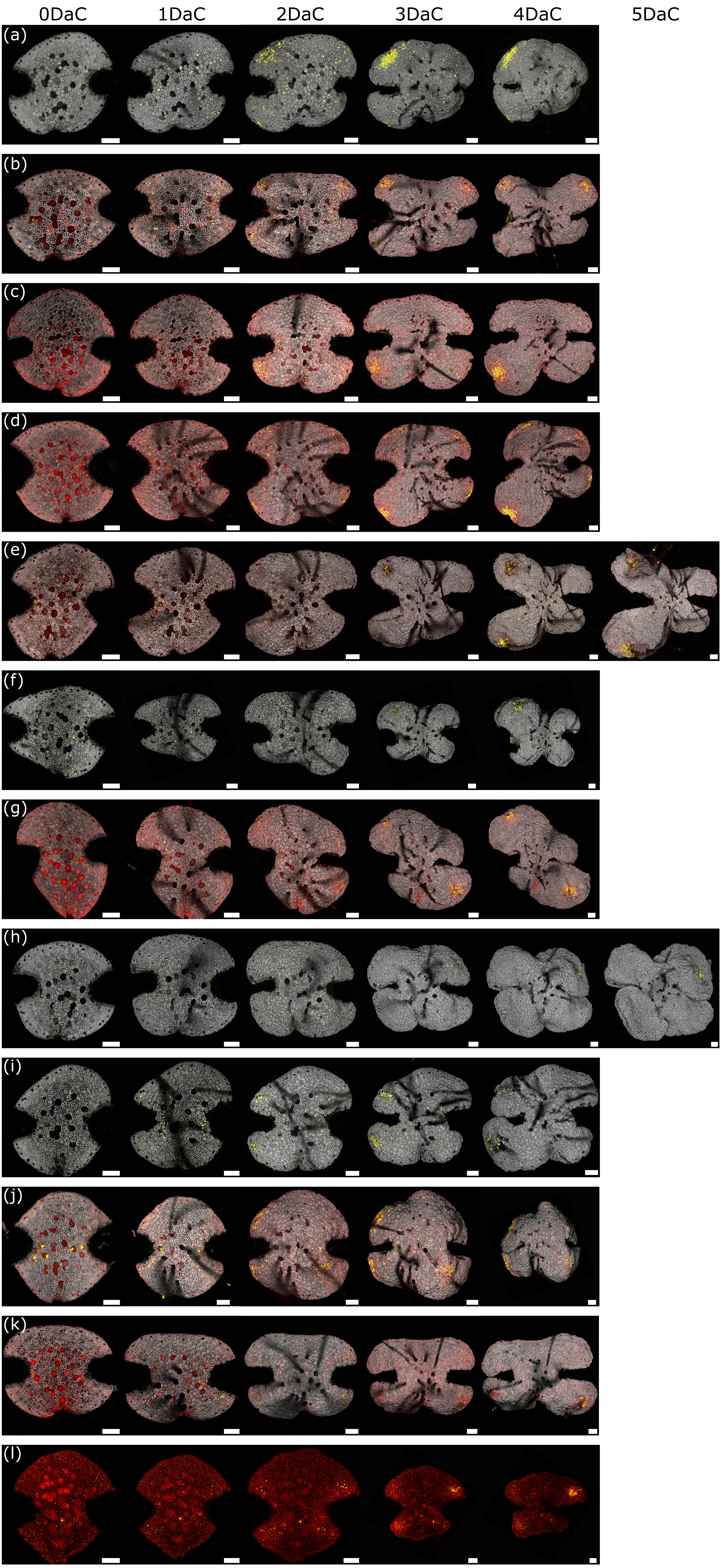

Figure S6 Timing of the reappearance of apical notch/meristem marker signal after excision of the apical notches.
(a) ET238-P25 imaged from 0DaC until 4DaC. (b) ET239-P14 imaged from 0DaC until 4DaC. (c) ET239-P21 imaged from 0DaC until 4DaC. (d) ET239-P33 imaged from 0DaC until 4DaC. (e) ET239-P75 imaged from 0DaC until 5DaC. (f) ET239-P82 imaged from 0DaC until 4DaC. (g) ET239-P125 imaged from 0DaC until 4DaC. (h) ET239-P127 imaged from 0DaC until 5DaC. (i) ET239-P133 imaged from 0DaC until 4DaC. (j) ET239-P153 imaged from 0DaC until 4DaC. (k) ET239-P156 imaged from 0DaC until 4DaC (chlorophyll channel included, cf. Figure 5(b)) (l) ET239-P161 imaged from 0DaC until 4DaC (chlorophyll channel omitted, cf. Figure 5 (a)). Scale bars= 100µm.

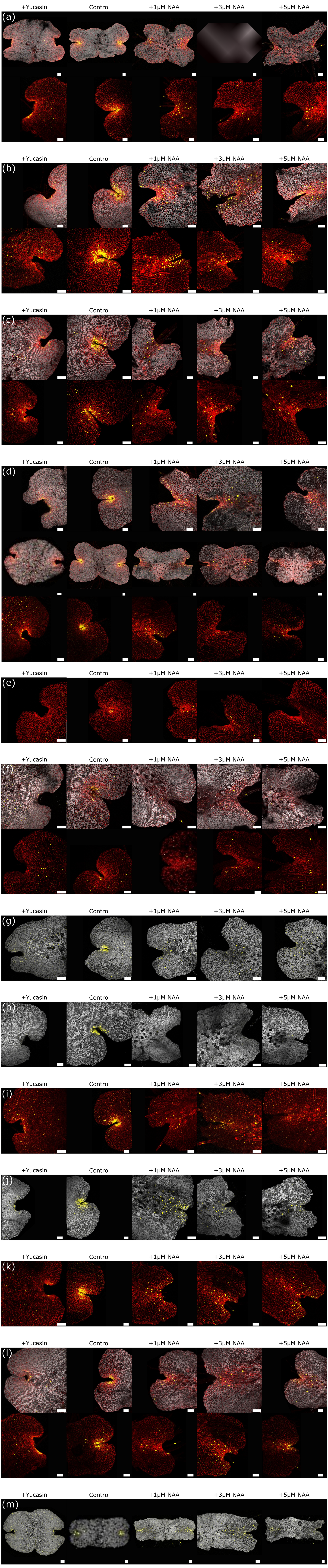

Figure S7. Auxin manipulation treatments of apical notch/meristem marker line gemmae. (a) ET239-P161 whole gemma images in top row, with chlorophyll channel omitted in images of a different gemma in bottom row. (b) ET239-P33; with chlorophyll channel omitted in images of a different gemma in bottom row. (c) ET239-P153; with chlorophyll channel omitted in images of a different gemma in bottom row. (d) ET239-P125; whole gemma images of a different gemma in middle row; chlorophyll omitted images of a different gemma in bottom row. The lines shown in (a)-(d) show signal distribution changes in respone to auxin manipulation that relates to changes in notch architecture.

(e) ET239-P156 with chlorophyll channel omitted. (f) ET239-P75; with chlorophyll channel omitted in images of a different gemma in bottom row. (g) ET239-P82. (h) ET239-P127. The lines shown in (e)-(h) have signal that is dramatically reduced or eliminated under elevated auxin treatments and auxin synthesis inhibitor treatment.

(i) ET239-P49 with chlorophyll channel omitted. (j) ET238-P25. The lines shown in (i) and (j) have reduced signal under elevated auxin treatments, whereas auxin synthesis inhibitor treatment causes changes in spatial distribution of signals.

(k) ET239-P21 with chlorophyll channel omitted. (l) ET239-P14; with chlorophyll channel omitted in images of a different gemma in bottom row. The lines shown in (k) and (l) has a response to elevated auxin treatments that relates to notch architecture, but auxin synthesis inhibitor treatment eliminates or markedly reduces signal.

(m) ET239-P133 whole gemma images. This line shows large spatial changes in signal distribution under both elevated auxin and inhibitor treatment, that appears to be unrelated to changes in notch architecture.

All gemma shown were imaged at 3dpg. In all sub-figures from left to right: +10μM yucasin, untreated control, +1μM NAA, +3μM NAA, +5μM NAA. Scale bars= 100μm.

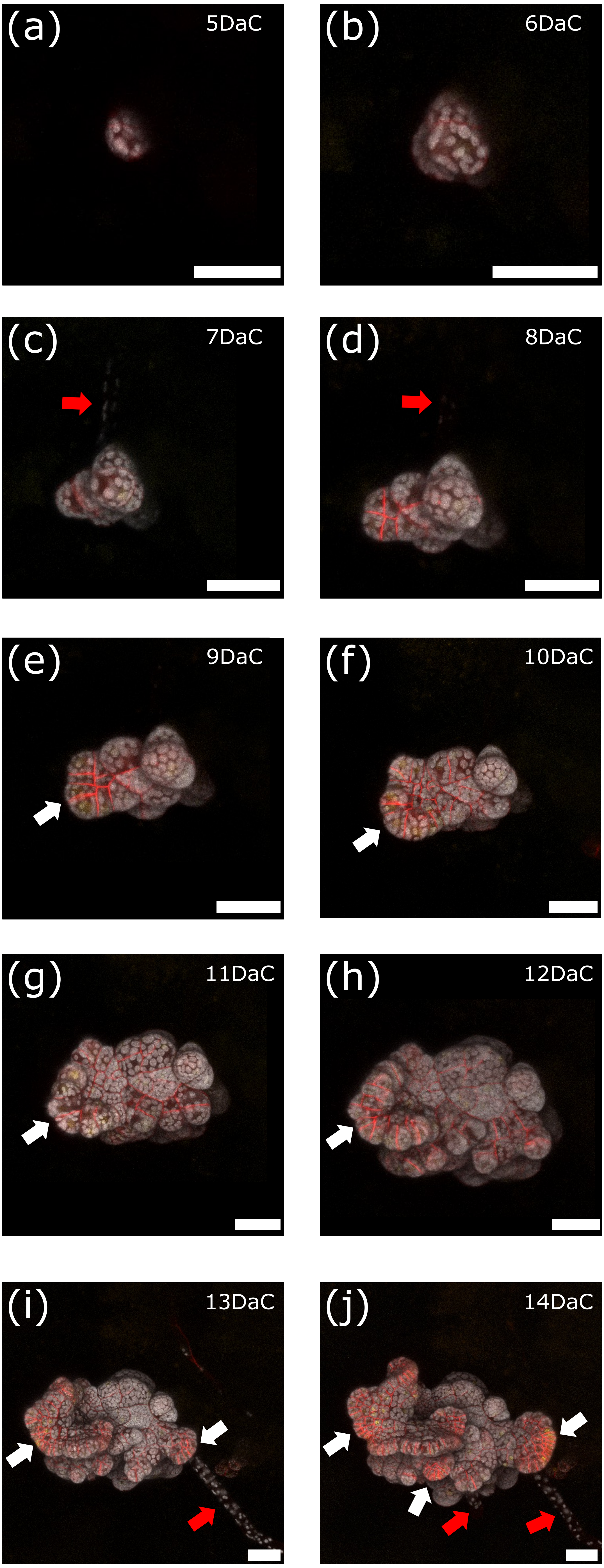

Figure S8 Developmental sequence of thallus regeneration from a small cluster of isolated gemma cells. Cells were isolated from the periphery of a 0DaC gemma from the apical notch/meristem marker line ET239-P161 by laser ablation. Images of the cells shown were taken daily from 5DaC (a) until 14DaC (j). Initially cell division proceeds with no marker signal apparent, to form a callus-like mass of photosynthetic cells with occasional rhizoids (red arrows). Marker signal appearance precedes and marks out the regenerating meristem region (white arrows) that will go on to form the apical notch of a new thallus. Scale bars= 50μm.

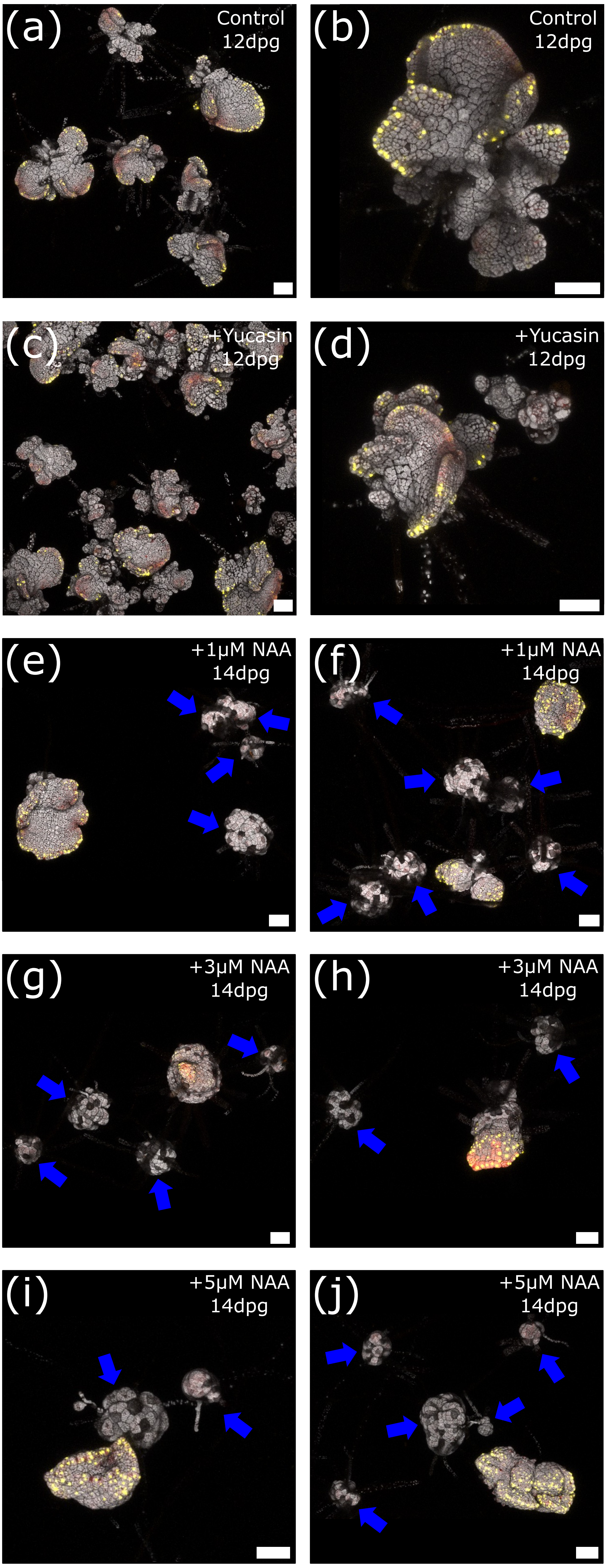

Figure S9 Effect of auxin manipulation on spore germination and development in margin tissue marker (ET239-P64) sporelings. (a), (b) Sporelings imaged 12dpg at the prothallus stage grown on control media. (c), (d) Sporelings imaged 12dpg at the prothallus stage grown on +10µM yucasin media. These show normal appearance of the prothallus and margin tissue marker signal. Sporelings imaged at 14dpg at the prothallus stage grown on +1µM NAA ((e), (f)), +3µM NAA ((g), (h)) and +5µM NAA ((i), (j)) media. Elevated auxin treatment retards sporeling development with prothalli taking longer to emerge, hence this stage not being observed until 14dpg. Even with the extended growth time most sporelings grown under elevated auxin did not proceed to the prothallus stage at all. Instead, these sporelings formed large callus-type protonema (blue arrows) and did not produce any margin tissue nor exhibit any margin tissue marker signal. Those sporelings that did proceed to that prothallus stage displayed elevated levels of margin tissue marker signal, correlated with the exogenous auxin concentration. This is similar to the situation in ET239-P64 gemma grown under elevated auxin levels (see Figure S3 (a)-(g)). Scale bars= 100µm.
